# Seasonal Malaria Chemoprevention with Sulphadoxine-Pyrimethamine and Amodiaquine Selects *Pfdhfr-dhps* Quintuple Mutant Genotype in Mali

**DOI:** 10.1371/journal.pone.0162718

**Published:** 2016-09-23

**Authors:** Hamma Maiga, Estrella Lasry, Modibo Diarra, Issaka Sagara, Amadou Bamadio, Aliou Traore, Samba Coumare, Soma Bahonan, Boubou Sangare, Yeyia Dicko, Nouhoum Diallo, Aly Tembely, Djibril Traore, Hamidou Niangaly, François Dao, Aboubecrine Haidara, Alassane Dicko, Ogobara K. Doumbo, Abdoulaye A. Djimde

**Affiliations:** 1 Malaria Research and Training Center, Department of Epidemiology of Parasitic Diseases, Faculty of Medicine and Odontostomatology and Faculty of Pharmacy, University of Sciences, Techniques and Technologies of Bamako, Mali; 2 Médecins Sans Frontières (MSF), New York, New York, United States of America; 3 Médecins Sans Frontières (MSF) Bamako, Mali; Johns Hopkins University Bloomberg School of Public Health, UNITED STATES

## Abstract

**Background:**

Seasonal malaria chemoprevention (SMC) with sulphadoxine-pyrimethamine (SP) plus amodiaquine (AQ) is being scaled up in Sahelian countries of West Africa. However, the potential development of *Plasmodium falciparum* resistance to the respective component drugs is a major concern.

**Methods:**

Two cross-sectional surveys were conducted before (August 2012) and after (June 2014) a pilot implementation of SMC in Koutiala, Mali. Children aged 3–59 months received 7 rounds of curative doses of SP plus AQ over two malaria seasons. Genotypes of *P*. *falciparum Pfdhfr* codons 51, 59 and 108; *Pfdhps* codons 437 and 540, *Pfcrt* codon 76 and *Pfmdr1*codon 86 were analyzed by PCR on DNA from samples collected before and after SMC, and in non-SMC patient population as controls (November 2014).

**Results:**

In the SMC population 191/662 (28.9%) and 85/670 (12.7%) of children were *P*. *falciparum* positive by microscopy and were included in the molecular analysis before (2012) and after SMC implementation (2014), respectively. In the non-SMC patient population 220/310 (71%) were successfully PCR analyzed. In the SMC children, the prevalence of all molecular markers of SP resistance increased significantly after SMC including the *Pfdhfr-dhps* quintuple mutant genotype, which was 1.6% before but 7.1% after SMC (p = 0.02). The prevalence of *Pfmdr1*-86Y significantly decreased from 26.7% to 15.3% (p = 0.04) while no significant change was seen for *Pfcrt* 76T. In 2014, prevalence of all molecular markers of SP resistance were significantly higher among SMC children compared to the non-SMC population patient (p < 0.01). No *Pfdhfr*-164 mutation was found neither at baseline nor post SMC.

**Conclusion:**

SMC increased the prevalence of molecular markers of *P*. *falciparum* resistance to SP in the treated children. However, there was no significant increase of these markers of resistance in the general parasite population after 2 years and 7 rounds of SMC.

## Introduction

Administering a full curative course of an antimalarial drug at set times regardless of parasitemia has been used as a malaria prevention tool known as Intermittent Preventive Treatment in infants [IPTi] and children [IPTc] [1]. Landmark studies demonstrated the protective values of both IPTi with sulphadoxine-pyrimethamine [SP] [2] and IPTc with SP and amodiaquine [AQ], which was renamed as Seasonal Malaria Chemoprevention [SMC] and recommended as a new policy by World Health Organization [WHO] [3]. However, both SP and AQ are known to select molecular markers of resistance, which may lead to increased in vivo resistance [3]. Amodiaquine resistance is associated with the molecular determinants of chloroquine resistance, the *Plasmodium falciparum* chloroquine resistance transporter [*Pfcrt*] and the *P*. *falciparum* multidrug resistance gene 1 [*Pfmdr1*] [4]. Resistance to sulphadoxine and pyrimethamine are due to point mutations in *P*. *falciparum* dihydropteroate synthase [*Pfdhps*] *and P*. *falciparum* dihydrofolate reductase [*Pfdhfr]*, respectively [5]. Unlike East and Southern Africa where SP and AQ resistance are widespread, SP plus AQ remain efficacious in West Africa [6]. The quintuple mutant genotype which include *Pfdhfr* [51I, 59R and 108N] and *Pfdhps* [437G and 540E] is known to be the most associated with in vivo SP resistance [7] while the mutations *Pfcrt* 76T and *Pfmdr1* 86Y are associated with AQ resistance [1]. IPTc with SP plus artesunate [AS] was shown to select the quadruple mutant genotype [*Pfdhfr* 51, 59 and 108 plus *Pfdhps* 437] in Senegal [7]. However, no such selection was found in similar interventions in Burkina Faso and Ghana [8]. The two most deleterious mutations for sulphadoxine resistance (*Pfdhps* 540) and pyrimethamine [*Pfdhfr* 164] were found during SMC studies neither in Senegal [9] nor in Burkina Faso [10]. Similar studies showed a higher prevalence of *Pfdhfr* and *Pfdhps* mutations in infections from children under SMC with SP plus AQ in Burkina Faso and Mali [11–13]. Most of the previous studies assessed the impact of SMC with SP plus AQ either immediately after the intervention or after just one season of SMC. The current study aims to assess the impact of SMC on the selection and spread of *Plasmodium* drug resistant parasites after several rounds of SMC.

## Methods

### Study site

This study was conducted in the health district of Koutiala, which has approximately 575,000 inhabitants and is located at 420 km south of Bamako, the capital city of Mali. Twenty rural health centers, all located within a radius of 15 km where randomly selected. Malaria transmission is hyper-endemic in the region with a sharp peak in transmission during the rainy season [April to November]. More than 80% of malaria cases occur between August and November (our unpublished data).

### Study design and participants

#### SMC population

Two cross-sectional surveys were conducted, one in August 2012 a few days before the start of the pilot SMC implementation and a second one in June 2014 i.e. 8 months after the last dose of SMC drugs were distributed. A cluster randomization was used to select the children. In each randomly selected cluster, households were also randomly selected to enroll at least 32 children per cluster [in the 2012 study] or 64 children per cluster [in the 2014 study] in the target age group. After identification of the children, informed consent was obtained from their parents prior to their interview and inclusion. Children were eligible to participate to the study if they were aged 3–59 months at the time of enrolment and residents of the study area with no intention to leave during the study period. Children with the following criteria were excluded: presence of a severe or chronic illness, such as severe malnutrition or Human immunodeficiency virus infection and acquired immunodeficiency syndrome [HIV/AIDS], and a history of a significant adverse reaction to SP or AQ. For all participants a thick blood smear and blood spot was collected by finger prick. In the 2012 malaria transmission season, three cycles of SMC with SP plus AQ were provided in August, September and October. In the malaria transmission season of 2013 four cycles of SMC with SP plus AQ were provided in July, August, September and October. SMC population included both symptomatic and asymptomatic children as long as they were within the age bracket of 3–59 months during the survey. The symptomatic cases were diagnosed with a Rapid diagnostic tests [RDT] [SD BIOLINE Malaria Ag P.f^®^, Suwon City, South Korea]; if found positive, they received artemether-lumefantrine [Coartem^R^] as per National Malaria Treatment Guidelines [NMTG].

#### Non-SMC patient population

In November 2014, to assess the potential flow of resistant genes in the neighboring general population we enrolled patients who had never received any round of SMC with SP plus AQ. Rapid diagnostic tests [RDT, SD BIOLINE Malaria Ag P.f^®^, Suwon City, South Korea] were dispatched by the study team to the health centers serving the clusters included in the SMC study. Patients visiting these health centers that were aged 7 years or above were offered free RDTs if they had any malaria symptoms. Those found to be malaria positive by RDT were treated immediately with artemether-lumefantrine [Coartem®] or artesunate-amodiaquine according to national guidelines. The used positive RDTs were collected and used to extract DNA for molecular analyses and included in the non-SMC patient population.

#### SMC delivery

SMC drugs were delivered by Médecins Sans Frontières, Bamako [MSF] in collaboration with the National Malaria Control Program of Mali. The delivery teams used a combination of fixed post [for small villages] and door-to-door strategy [for large villages]. The drug resistance study team was not involved in the delivery of SMC drugs.

### Assessment of molecular markers of *P*. *falciparum* resistance to sulphadoxine, pyrimethamine and amodiaquine

DNA was extracted from selected dried blood spots [DBS] on filter papers (3MM Whatman) as described [14]. Assessment of drug resistance markers was performed by nested PCR followed by restriction digestion [14–16]. Drug resistance makers tested for this study were *Pfdhfr* N51I, *Pfdhfr* C59R, *Pfdhfr* S108N, *Pfdhfr* I164L for pyrimethamine, *Pfdhps* A437G and *Pfdhps* K540E for sulphadoxine, *Pfcrt* K76T and *Pfmdr1* N86Y for amodiaquine. Results were classified as wild type, mutant or mix [when both alleles were present]. Cases of mix infection were categorized as mutants.

### Sample size

Based on previous studies conducted in Mali, the prevalence of quintuple mutation was zero before SMC [Prevalence (P1) = 0.0%] [12]. We estimate a prevalence of 10% after two years of SMC implementation [P2 = 10.0%] in 2014. To estimate our sample size we set alpha at 5 with a confidence interval of 95% and beta at 10% with a power of 90%. We define Zα as the z value for risk of error α [α = 0.05, z = 1.96 two-tailed test], Zβ as the z value for risk of β error [1-β = 0.90, z = 1.282] and Δ detection to be 10%. Based on these criteria and using a Poisson approximation, to detect a change from 0.0% to 10.0% in prevalence of quintuple mutation genotype with a power of 90%, the sample size was estimated to 105 subjects per time period [total 210 subjects]. With an estimated 10% of excluded samples, we will need 234 subjects [117 per time period]. Also considering that the prevalence of malaria is 50% in Koutiala, we expect to have 1 out of 2 samples to be positive for malaria. Therefore the total number of volunteers was estimated at 468. Since 10% of dried blood spots may not be usable, the sample size was set at 515 volunteers.

### Data management and analysis

Data were collected on case report forms, double entered, and analyzed with MS Access and/or Stata [Stata Corp 11]. Prevalence of single mutations and of various genotypes including the double mutant [*Pfcrt* 76 + *Pfmdr1* 86], triple mutant [*Pfdhfr* 51 + 59 + 108], quadruple mutant [triple *Pfdhfr* mutant + *dhps* 437], quintuple mutant [quadruple mutant + *dhps* 540], six mutation genotype with *Pfcrt* [*Pfdhfr-dhps* quintuple + *Pfcrt*-76T] or *Pfmdr1 [Pfdhfr-dhps* quintuple + *Pfmdr1*-86Y] and seven mutations genotype [*Pfdhfr-dhps* quintuple + *Pfcrt*-76T + *Pfmdr1*-86Y] were calculated with 95% Confidence interval [CI]. Chi-square or Fisher exact probability tests were used for comparisons as appropriate with statistical significance set at *P value* < 0.05.

### Ethics

The study protocol was approved by the Ethical Committee of the Faculty of Medicine and Odonto-stomatology (FMOS) and Faculty of Pharmacy (FAPH)/ University of Sciences, Techniques and Technologies of Bamako [USTTB]. Community permission was obtained from each locality prior to the study. Individual, written, informed consent was obtained from parents or guardians of each child prior to screening.

## Results

### Demographic characteristics

The trial profile is summarized in Table 1. From 4 to 9 August 2012 and 20 to 30 June 2014, 662 and 670 children aged from 3 to 59 months were enrolled in the study, respectively. The children median age and sex ratio of female proportion were 2 years and 54.5% in baseline 2012 and 3 years and 49.7% in post intervention 2014, respectively. The proportion of fever, anaemia and *P*. *falciparum* presence was 6.5%, 67.5% and 28.9% in baseline and 2.4%, 48.5% and 12.7% in post intervention, respectively. In the non-SMC patient population with 502 patients, the median age was 20 years with 60.6% for sex ratio of female and the proportion of history of fever and fever were 79.9% and 50%, respectively [S1, S3 and S5 Files].

**Table 1:** Demographic, clinical, and laboratory participant characteristics in SMC and non-SMC population.

	SMC population 2012 (N = 662)		SMC population 2014 (N = 670)		NON-SMC population 2014 (N = 502)	
	n	%/median	n	%/median	n	%/median
Sex ratio (female)	361	54.5%	333	49.7%	304	60.6%
Age	661	2 years	670	3 years	502	20 years
Urban	192	28.7%	190	28.4%	150	29.9%
History of fever	213	32.2%	104	15.5%	401	79.9%
Fever	43	6.5%	16	2.4%	251	50%
*P*. *falciparum*	190	28.8%	83	12.4%	-	-
*P*. *malariae*	2	0.3%	3	0.5%	-	-
*P*. *ovale*	3	0.5%	0	0%	-	-
Gametocyte	60	9.1%	15	2.2%	-	
TDR (+)	43	6.5%	16	2.4%	359	71.5%
Haemoglobin <11g/dl	446	67.5%	336	50.2%	-	

RTD, Rapid diagnostic test; n, number with positive; N, number of participants.

### Molecular markers of antimalarial drug resistance

In the SMC population 191/662 [28.9%] and 85/670 [12.7%] of children were *P*. *falciparum* positive by microscopy and were included in the molecular analysis before [2012] and after SMC implementation [2014], respectively. Among the 502 volunteers recruited in 2014 in the non-SMC patient population, 310/502 [71.5%] had a positive RDT. Upon nested PCR, 220/310 [71.6%] of these RDT positive samples yielded DNA amplification Table 1.

Table 2 shows the prevalence of molecular markers associated with resistance to sulphadoxine, pyrimethamine and amodiaquine at baseline and after SMC intervention in the SMC children. The prevalence of *Pfdhfr* 51I, 59R and 108N [92.1% to 100%, p = 0.01] and *Pfdhps* 540E [3.1% to 10.6%, p = 0.01] were significantly higher in children after SMC intervention compared to the baseline. There was a non-significant increase in *Pfdhps* 437G from baseline to post intervention [61.3% to 70.6%, p = 0.14]. The prevalence of *Pfdhfr* triple, *Pfdhfr-dhps* quadruple and *Pfdhfr-dhps* quintuple mutants at baseline *versus* after intervention were 92.6% *versus*. 100% [p = 0.01], 57.9% *vs*. 75.3% OR = 1.77 [95% confidence interval (CI) 0.99–3.20] [p = 0.04] and 1.6% vs. 7.1% OR = 4.76 [95% CI 0.98–29.94] [p = 0.02], respectively. Prevalence of *Pfmdr1*-86Y, *Pfcrt*-76T and double mutant *Pfmdr1*-86Y + *Pfcrt*-76T before *vs*. after intervention were 26.7% *vs*. 15.3% [p = 0.04]; 68.1% *vs*. 75.3% [p = 0.22] and 18.8% *vs*. 10.6% [p = 0.09], respectively. No mutation at codon *Pfdhfr* 164 was found neither at baseline nor post SMC intervention (S2 and S4 Files).

**Table 2:** Prevalence of molecular markers of resistance to sulphadoxine, pyrimethamine and amodiaquine at baseline and post-SMC in the SMC population.

Genes	Baseline 2012		Post intervention 2014			
	n/N	% Mutant	n/N	% Mutant	Odds Ratio [OR] 95%CI	P-value
*Pfdhfr* 51	176/191	92.1	85/85	100	-	0.01
*Pfdhfr* 59	176/191	92.1	85/85	100	-	0.01
*Pfdhfr* 108	176/191	92.1	85/85	100	-	0.01
Triple *Pfdhfr* mutants	176/191	92.1	85/85	100	-	0.01
*Pfdhps* 437	117/191	61.3	60/85	70.6	1.52 (0.85–2.75)	0.14
*Pfdhps* 540	6/191	3.1	9/85	10.6	3.65 (1.11–12.85)	0.01
Quadruple mutants (triple *Pfdhfr* + *dhps* 437)	110/191	57.6	64/85	75.3	1.77 (0.99–3.20)	0.04
Quintuple mutants (quadruple mutants + *dhps* 540)	3/191	1.6	6/85	7.1	4.76 (0.98–29.94)	0.02
*Pfcrt* 76	130/191	68.1	64/85	75.3	1.43 (0.78–2.69)	0.22
*Pfmdr1*- 86	51/191	26.7	13/85	15.3	0.50 (0.23–1.00)	0.04
Double mutants (*Pfcrt* 76 + *Pfmdr1*- 86)	36/191	18.8	9/85	10.6	0.51 (0.21–1.15)	0.09

n, number with mutant; N, number of participants with parasitaemia at blood smear tested.

When focusing on 2014, the prevalence of the following mutations were higher in the SMC population than in non-SMC patient population: *Pfdhfr* 51I, 100% *vs*. 81.7%; *Pfdhfr* 59R, 100% *vs*. 87.0%; *Pfdhfr* 108N, 100% *vs*. 81.4%; and *Pfdhps*-540, 10.6% *vs*. 4.1% [p < 0.05 for each comparison] Table 3. Similarly, the prevalence of *Pfdhfr* triple [100% *vs*. 64.9%], *Pfdhfr-dhps* quadruple [75.3% vs. 43.6%] and *Pfdhfr-dhps* quintuple mutant genotypes [7.1% to 0.46%] were significantly higher in the SMC population as compared to non-SMC patient population [p < 0.01 for each comparison]. There were no statistically significant differences with the remaining genotypes and *Pfdhfr* 164 mutation was not detected in the study (S4 and S6 Files).

**Table 3:** Prevalence of molecular markers of resistance to sulphadoxine, pyrimethamine and amodiaquine at post-SMC in the SMC population *vs*. concurrent Non-SMC patient population.

Genes	Post-SMC population 2014		Non-SMC patient population 2014			
	n/N	% Mutant	n/N*	% Mutant	Odds Ratio 95%CI	P-value
*Pfdhfr* 51	85/85	100	165/202	81.7	--	<0.001
*Pfdhfr* 59	85/85	100	167/192	87.0	--	<0.001
*Pfdhfr* 108	85/85	100	144/177	81.4	--	<0.001
Triple *Pfdhfr* mutants	85/85	100	109/168	64.9	--	<0.001
*Pfdhps* 437	60/85	70.6	125/177	70.6	--	>0.05
*Pfdhps* 540	9/85	10.6	9/220	4.1	2.78(0.93–8.19)	0.03
Quadruple mutants (triple *Pfdhfr* + *dhps* 437)	64/85	75.3	71/163	43.6	3.95(2.13–7.44)	<0.001
Quintuple mutants (quadruple mutants + *Pfdhps* 540)	6/85	7.1	1/216	0.46	16.33(1.9–754.34)	0.006
*Pfcrt 76*	64/85	75.3	94/132	71.2	1.23(0.64–2.42)	0.51
*Pfmdr1*- 86	13/85	15.3	21/99	21.2	0.67(0.29–1.53)	0.30
Double mutants (*Pfcrt* 76 + *Pfmdr1*- 86)	9/85	10.6	15/106	14.2	0.71(0.26–1.87)	0.46

n, number with mutant; N, number of participants with parasitaemia at blood smear tested

N*,number of participants with positive at Rapid diagnostic test.

The prevalence of molecular markers in the baseline children and the non-SMC patient population [7 years of age or older] is shown in Table 4. Prevalence of *Pfdhfr* 51I [92.1% to 81.7%] and *Pfdhfr* 59R [92.1% to 81.4%], [p = 0.001] were higher at baseline and post-intervention in the SMC population than in the non-SMC patient population, respectively. The prevalence of triple and quadruple mutants were significantly higher in the SMC children at baseline and post-intervention than in the non-SMC patient population with 92.6% *vs*. 64.9% [p < 0.001] and 57.9% *vs*. 40.1% [p = 0.009], respectively. There were no statistically significant differences with the remaining codons. Mutation at codon 164 of *Pfdhfr* was not detected (S2 and S6 Files).

**Table 4:** Prevalence of molecular markers of resistance to sulphadoxine, pyrimethamine and amodiaquine at baseline and post intervention period in Non-SMC patient population.

Genes	Baseline children 2012		Non-SMC patient population 2014			
	n/N	% Mutant	n/N*	% Mutant	Odds Ratio 95%CI	P-value
*Pfdhfr* 51	176/191	92.1	165/202	81.7	2.82 (1.42–5.85)	0.001
*Pfdhfr* 59	176/191	92.1	167/192	87.0	1.88 (0.90–4.05)	0.07
*Pfdhfr* 108	176/191	92.1	144/177	81.4	2.88 (1.43–6.05)	0.001
Triple *Pfdhfr* mutants	176/191	92.1	109/168	64.9	6.80 (3.53–13.78)	<0.001
*Pfdhps* 437	117/191	61.3	125/177	70.6	0.66 (0.42–1.04)	0.06
*Pfdhps* 540	6/191	3.1	9/220	4.1	0.76 (0.22–2.45)	0.61
Quadruple mutants (triple *Pfdhfr* + *dhps* 437)	110/191	57.9	71/163	43.6	1.76 (1.13–2.74)	0.009
Quintuple mutants (quadruple mutants + *Pfdhps* 540)	3/191	1.6	1/216	0.46	3.43 (0.27–180.91)	0.26
*Pfcrt* 76	130/191	68.1	94/132	71.2	0.99 (0.51–1.44)	0.55
*Pfmdr1*- 86	51/191	26.7	21/99	21.2	1.35 (.74–2.55)	0.30
Double mutants (*Pfcrt* 76 + *Pfmdr1*- 86)	36/191	18.8	15/106	14.2	1.41 (0.71–2.93)	0.30

n, number with mutant; *N*, number of participants with parasitaemia at blood smear tested

N*, number of participants with positive at Rapid diagnostic test

The prevalence of six-mutation genotype with *Pfmdr1* [*Pfdhfr-dhps* quintuple + *Pfmdr1*-86Y] was 1.2%, six-mutation genotype with *Pfcrt* [*Pfdhfr-dhps* quintuple + *Pfcrt*-76T] was 7.1% and seven-mutation genotype [*Pfdhfr-dhps* quintuple + *Pfmdr1*-86Y + *Pfcrt*-76T] was 1.2% among SMC-children post-intervention in 2014. No six-mutation and seven-mutation genotypes were observed neither among SMC children at baseline nor in the control non-SMC patient population [data not shown].

## Discussion

We show for the first time the presence of the *Pfdhfr* and *Pfdhps* quintuple mutant genotypes in a population that received SMC with sulphadoxine-pyrimethamine plus amodiaquine over two malaria seasons in West Africa. Furthermore, there was a four-fold increase in this quintuple mutant genotype after seven rounds of SMC in this setting. This is particularly worrisome since *Pfdhfr-dhps* quintuple mutant genotype was shown to be the most associated with in *vivo* failure of sulphadoxine-pyrimethamine in the treatment of uncomplicated *falciparum* malaria [17]. Nevertheless, the nearly 10% of quintuple mutant genotype found in our post-SMC population is still significantly lower than rates of this genotype in other malaria endemic regions of Africa [18–20]. Similar to previous studies in West Africa, we found a significant selection of individual *Pfdhfr* [codons 51, 59, 108] and *Pfdhps* [codons 437, 540] mutations in children enrolled in the SMC program. As a consequence the frequencies of the *Pfdhfr* triple mutant genotype and the *Pfdhfr-dhps* quadruple mutant genotype were significantly higher in the post-SMC. In addition, the post-SMC prevalence found in this study was higher than post-SMC prevalence of *Pfdhfr* [codons 51, 59, 108] and *Pfdhps* [codons 437, 540] mutations found in other settings of Mali such as Djoliba, Siby and Ouelessebougou [12] as well as in neighboring countries of Senegal and Burkina Faso [7, 10, 13]. However, the significant post-SMC selection of the *Pfdhfr* triple mutation and the *Pfdhfr*-*dhps* quadruple mutation observed in this study is consistent with results observed after two years of implementation of intermittent preventive treatment [IPTi] with SP alone in infants in Gabon, Cameroun and Senegal [21–23]. Compared to other studies in Mali and in West Africa, this study did show high baseline levels of individual and triple *Pfdhfr* mutant genotypes of 92.6% [14–16, 24]. This may reflect the long history of usage of sulpha drugs including SP in our study site. Although the *Pfdhfr* triple mutation was prevalent in more than 90% of infections, it is well established that this genotype does not correlate with *in vivo* SP failure in West Africa [16, 24]. The prevalence of *Pfdhfr-dhps* quadruple mutant genotype was higher than the prevalence of that genotype in several West African sites [16, 24–26].

We found *Pfdhps* 540E in 3% of baseline infections. There is an upward trend of the prevalence of *Pfdhps* 540E in Mali, as this mutation was not detected at baseline in a study conducted in 2008 Mali [2]. Our rates were similar to findings from Ghana [27] but lower than those of a refugee camp in Guinea [28]. Mutations at codon 540 of *Pfdhps* and codon 164 of *Pfdhfr* were not detected in a study conducted in three health districts in Senegal with 54 health posts with a gradual introduction of SMC [9].

*Pfcrt* and *Pfmdr1* prevalence were similar to previous reports from Mali [12, 14], Burkina Faso [10] and Senegal [9]. The pre-SMC and post-SMC rates of *Pfcrt* 76T and *Pfcrt* 76T + *Pfmdr1* 86Y were comparable. However, there was a significant decrease of the prevalence of *Pfmdr1* 86Y in post-SMC patients, which may be due to the counter selection exerted by lumefantrine on this mutation [29–31] The effects of SP plus AQ on molecular markers of resistance to AQ require further investigation. A large body of literature shows that after malaria treatment with regimens that included AQ subsequent infections showed selection of parasites with *Pfcrt* and *Pfmdr1* polymorphisms associated with AQ resistance [6, 14, 32, 33] and with decreased *in vitro* AQ sensitivity [34]. This appears to not be case when SP plus AQ is used.

We found that 8 months after SMC, children in the SMC age bracket had a higher prevalence of molecular markers of SP resistance than the non-SMC patient population, even though the two populations were living together in the same areas. This observation is indeed intriguing and we have no clear explanation for it. However, previous reports showed *in vitro* that parasites exhibiting pyrimethamine-resistant phenotype appear throughout the 54-day period following SP treatment [35]. We also showed seventeen years ago that following a single dose of sulphadoxine-pyrimethamine oral treatment, *Pfdhfr* mutations were selected up to 53 days later [36]. Given that these studies were conducted when SP was nearly fully susceptible *in vivo* as shown by the low prevalence of *Pfdhfr* and *Pfdhps* mutations, one could speculate that this selective pressure could last several months when the starting parasite population has much higher rates of these mutations as is the case now. In addition, the seven rounds of SMC with SP plus AQ given in this study could lead to drug accumulation in the treated population, further prolonging the elimination half-lives of the respective drugs and resulting in selection of molecular markers 8 months after the last round of SMC. Additional parasites genetic studies and drug pharmacokinetics studies will be required to clarify these issues.

To assess the potential spread of drug resistance genes in the general parasite population from the SMC areas, we measured the prevalence of molecular markers of resistance to the SMC drugs in contemporaneous populations that were not eligible for SMC because they were older than 5 years of age. Both SMC and non-SMC participants were from the same villages of the Health District of Koutiala although the former group was aged between 3 and 59 months while the latter group was aged 7 years and above. Although most of the SMC children were asymptomatic, some [approximately 5%] were symptomatic during the survey period while all of the non-SMC participants were clinical malaria cases. Interestingly, despite the significant selection of molecular markers of *P*. *falciparum* resistance to SP in the population that received the SMC regimens, there was no such increase in the general parasite population from the same study areas. This observation is consistent with previous reports from Mali [12] and in the sub-Region [10]. We reckon that no age matching was done because we purposely had to choose older children so as to exclude patients exposed to SMC. However, both the SMC group and the non-SMC patient populations were from the same exact villages and were therefore exposed to the same malaria transmission conditions and to the same pool of malaria parasites. Nevertheless, because of the difference in age between the two groups, differences in immunity could play a role in the prevalence of parasitaemia and/or in the clearance of mutant parasites [37]. Yet, no difference in prevalence of markers of resistance was found between younger and older patients in a study in Benin, neither in symptomatic patients nor in asymptomatic ones [38].

Although SMC appears to have contributed to the increase in frequency of molecular markers of SP resistance the overall efficacy of SP plus AQ may not be compromised at this stage. Indeed, (i) there was no significant increased prevalence of markers of resistance to AQ resistance in the general population, (ii) there was no spread of the markers of SP resistance in the general parasite population in the same area, and (iii) the rate of *Pfdhps* 540E at baseline is still at 3%, much lower than the WHO threshold of 50% that is thought to compromise SP efficacy in intermittent preventive treatment. Indeed, a WHO technical advisory group recommended that in countries with a greater than 50% prevalence of the mutation at *Pfdhps* 540, IPTi with SP should not be initiated or continued [39] and the expected *in vivo* failure rate of a 10% quintuple mutant would be 5% [17].

## Conclusion

SMC with SP plus AQ remains warranted in this setting but, the increasing trend of the prevalence of *Pfdhps* 540E and the significant increase in the prevalence of *Pfdhfr-dhps* quintuple mutant genotype in the treated population several months after the last SMC round call for close monitoring of the efficacy of these SMC drugs as well as an acceleration of the development of replacement regimens.

## Supporting Information

S1 File SMC population 2012 Demographic Data. (PDF)

S2 File SMC population 2012 PCR Data. (PDF)

S3 File SMC population 2014 Demographic Data. (PDF)

S4 File SMC population 2014 PCR Data. (PDF)

S5 File Non-SMC population 2014 Demographic Data. (PDF)

S6 File Non-SMC population 2014 PCR Data. (PDF)

## References

[1] World Health Organization. World Malaria Report. Nature. 2013. doi:ISBN 978 92 4 1564403.

[2] Aponte JJ, Schellenberg D, Egan A, Breckenridge A, Carneiro I, Critchley J, et al Efficacy and safety of intermittent preventive treatment with sulfadoxine-pyrimethamine for malaria in African infants: a pooled analysis of six randomised, placebo-controlled trials. Lancet. 2009;374, 1533–1542. 10.1016/S0140-6736(09)61258-7 19765816

[3] World Health Organization (WHO) policy recommendation: seasonal malaria chemoprevention (SMC) for Plasmodium falciparum malaria control in highly seasonal transmission areas of the Sahel sub-region in Africa WHO, 2012; Geneva, Switzerland.

[4] Ecker A, Lehane AM, Clain J, Fidock DA. PfCRT and its role in antimalarial drug resistance. Trends Parasitol. 2012;28:504–514. 10.1016/j.pt.2012.08.002 23020971 PMC3478492

[5] Gregson A, Plowe CV. Mechanisms of resistance of malaria parasites to antifolates. Pharmacol. Rev. 2005;57:117–145. 15734729 10.1124/pr.57.1.4

[6] Zongo I, Dorsey G, Rouamba N, Tinto H, Dokomajilar C, Guiguemde RT, et al Artemether-lumefantrine versus amodiaquine plus sulfadoxine-pyrimethamine for uncomplicated falciparum malaria in Burkina Faso: a randomised non-inferiority trial. Lancet. 2007;369: 491–498. 17292769 10.1016/S0140-6736(07)60236-0

[7] Cissé B, Sokhna C, Boulanger D, Milet J, Bâ el H, Richardson K, et al Seasonal intermittent preventive treatment with artesunate and sulfadoxine-pyrimethamine for prevention of malaria in Senegalese children: A randomised, placebo-controlled, double-blind trial. Lancet. 2006;367, 659–667. 16503464 10.1016/S0140-6736(06)68264-0

[8] Kweku M, Liu D, Adjuik M, Binka F, Seidu M, Greenwood B, et al Seasonal intermittent preventive treatment for the prevention of anaemia and malaria in Ghanaian children: a randomized, placebo controlled trial. PLoS ONE. 2008;3(12): e4000 10.1371/journal.pone.0004000 19098989 PMC2602973

[9] Lo AC, Faye B, Ba el-H, Cisse B, Tine R, Abiola A, et al Prevalence of molecular markers of drug resistance in an area of seasonal malaria chemoprevention in children in Senegal. Malar J. 2013;12: 137 10.1186/1475-2875-12-137 23617576 PMC3652725

[10] Somé AF, Zongo I, Compaoré YD, Sakandé S, Nosten F, Ouédraogo JB, et al Selection of Drug Resistance-Mediating Plasmodium falciparum Genetic Polymorphisms by Seasonal Malaria Chemoprevention in Burkina Faso. Antimicrob Agents Chemother. 2014;58(7) 3660–5. 10.1128/AAC.02406-14 24733476 PMC4068591

[11] Zongo I, Milligan P, Compaore YD, Some AF, Greenwood B, Tarning J, et al Randomized Non-inferiority Trial of Dihydroartemisinin-Piperaquine Compared with Sulfadoxine-Pyrimethamine plus Amodiaquine for Seasonal Malaria Chemoprevention in Burkina Faso. Antimicrob Agents Chemother. 2015;59(8): 4387–96. 10.1128/AAC.04923-14 25918149 PMC4505196

[12] Dicko A, Diallo AI, Tembine I, Dicko Y, Dara N, Dicko Y, et al Intermittent Preventive Treatment of Malaria Provides Substantial Protection against Malaria in Children Already Protected by an Insecticide-Treated Bednet in Mali: A Randomised, Double-Blind, Placebo-Controlled Trial. PLoS Med. 2011;8(2): e1000407 10.1371/journal.pmed.1000407 21304923 PMC3032550

[13] Konate´ AT, Yaro JB, Ouedraogo AZ, Diarra A, Gansané A, Soulama I, et al Intermittent preventive treatment of malaria provides substantial protection against malaria in children already protected by an insecticide-treated bednet in Burkina Faso: a randomized, double blind, and placebo controlled trial. PLoS Med. 2011;8(2): e1000408 10.1371/journal.pmed.1000408 21304925 PMC3032552

[14] Djimdé AA, Fofana B, Sagara I, Sidibe B, Toure S, Dembele D, et al Efficacy, safety, and selection of molecular markers of drug resistance by two ACTs in Mali. Am. J. Trop. Med. Hyg. 2008;78, 455–461. 18337343

[15] Ndiaye D, Daily JP, Sarr O, Ndir O, Gaye O, Mboup S, et al Defining the origin of Plasmodium falciparum resistant dhfr isolates in Senegal. Acta Trop. 2006;99, 106–111 16905111 10.1016/j.actatropica.2006.07.002 PMC2582374

[16] Tinto H, Ouédraogo JB, Zongo I, van Overmeir C, van Marck E, Guiguemdé TR, et al Sulfadoxine-pyrimethamine efficacy and selection of Plasmodium falciparum DHFR mutations in Burkina Faso before its introduction as intermittent preventive treatment for pregnant women. Am. J. Trop. Med. Hyg. 2007;76, 608–613. 17426157

[17] Kublin JG, Dzinjalamala FK, Kamwendo DD, Malkin EM, Cortese JF, Martino LM, et al Molecular markers for failure of sulfadoxine-pyrimethamine and chlorproguanil-dapsone treatment of Plasmodium falciparum malaria. J Infect Dis. 2002;185(3):380–8. 11807721 10.1086/338566

[18] Shah M, Omosun Y, Lal A, Odero C, Gatei W, Otieno K, et al Assessment of molecular markers for anti-malarial drug resistance after the introduction and scale-up of malaria control interventions in western Kenya. Malar J. 2015;14: 75 10.1186/s12936-015-0588-4 25889220 PMC4331436

[19] Iriemenam NC, Shah M, Gatei W, van Eijk AM, Ayisi J, Kariuki S, et al Temporal trends of sulphadoxine-pyrimethamine (SP) drug-resistance molecular markers in Plasmodium falciparum parasites from pregnant women in western Kenya. Malar J. 2012;11:134 10.1186/1475-2875-11-134 22540158 PMC3390272

[20] Mbugi EV, Mutayoba BM, Malisa AL, Balthazary ST, Nyambo TB, Mshinda H. Drug resistance to sulphadoxine-pyrimethamine in Plasmodium falciparum malaria in Mlimba, Tanzania. Malar J. 2006;5:94 17076899 10.1186/1475-2875-5-94 PMC1636063

[21] Aubouy A, Jafari S, Huart V, Migot-Nabias F, Mayombo J, Durand R, et al DHFR and DHPS genotypes of Plasmodium falciparum isolates from Gabon correlate with in vitro activity of pyrimethamine and cycloguanil, but not with sulfadoxine-pyrimethamine treatment efficacy. J Antimicrob Chemother. 2003;52(1):43–9. 12805261 10.1093/jac/dkg294

[22] Nfor ENMW, Njifutri N, Mbulli IA, Tawé B, Masumbi NP, Venyoka A. Evaluation de la chimiorésistance à la sulfadoxine-pyrimethamine et des mutations des genes dhfr et dhps dans le district de sante de Ndu au nord-ouest Cameroun. Afr J Sci Tech. 2007;8, 52–55.

[23] Faye B, Ndiaye M, Ndiaye JL, Annie A, Tine RC, Lo AC, et al Prevalence of molecular markers of Plasmodium falciparum resistance to sulfadoxine-pyrimethamine during the intermittent preventive treatment in infants coupled with the expanded program immunization in Senegal. Parasitol Res. 2011;109(1):133–8. 10.1007/s00436-010-2236-9 21207062

[24] Mockenhaupt FP, Teun Bousema J, Eggelte TA, Schreiber J, Ehrhardt S, Wassilew N, et al Plasmodium falciparum dhfr but not dhps mutations associated with sulphadoxine-pyrimethamine treatment failure and gametocyte carriage in northern Ghana. Trop Med Int Health. 2005;10(9):901–8. 16135198 10.1111/j.1365-3156.2005.01471.x

[25] Dokomajilar C, Lankoande ZM, Dorsey G, Zongo I, Ouedraogo JB, Rosenthal PJ. Roles of specific Plasmodium falciparum mutations in resistance to amodiaquine and sulfadoxine-pyrimethamine in Burkina Faso. Am J Trop Med Hyg. 2006;75(1):162–5. 16837725

[26] Diallo DA, Sutherland C, Nebie I, Konate AT, Ord R, Ilboudo-Sanogo E, et al Children in Burkina Faso who are protected by insecticide-treated materials are able to clear drug-resistant parasites better than unprotected children. J Infect Dis. 2007;196(1):138–44. 17538894 10.1086/518252

[27] Marks F, Evans J, Meyer CG, Browne EN, Flessner C, von Kalckreuth V, et al High prevalence of markers for sulfadoxine and pyrimethamine resistance in Plasmodium falciparum in the absence of drug pressure in the Ashanti region of Ghana. Antimicrob Agents Chemother. 2005;49(3):1101–5. 15728909 10.1128/AAC.49.3.1101-1105.2005 PMC549270

[28] Bonnet M, Roper C, Félix M, Coulibaly L, Kankolongo GM, Guthmann JP. Efficacy of antimalarial treatment in Guinea: in vivo study of two artemisinin combination therapies in Dabola and molecular markers of resistance to sulphadoxine-pyrimethamine in N'Zérékoré. Malar J. 2007;6:54 17477865 10.1186/1475-2875-6-54 PMC1868032

[29] Sisowath C, Petersen I, Veiga MI, Mårtensson A, Premji Z, Björkman A, et al In vivo selection of Plasmodium falciparum parasites carrying the chloroquine-susceptible pfcrt K76 allele after treatment with artemether-lumefantrine in Africa. J Infect Dis. 2009;199(5):750–7. 10.1086/596738 19210165 PMC2718568

[30] Achieng AO, Muiruri P, Ingasia LA, Opot BH, Juma DW, Yeda R, et al Temporal trends in prevalence of Plasmodium falciparum molecular markers selected for by artemether-lumefantrine treatment in pre-ACT and post-ACT parasites in western Kenya. Int J Parasitol Drugs Drug Resist. 2015;5(3):92–9. 10.1016/j.ijpddr.2015.05.005 26236581 PMC4501530

[31] Kiaco K, Teixeira J, Machado M, do Rosário V, Lopes D. Evaluation of artemether-lumefantrine efficacy in the treatment of uncomplicated malaria and its association with pfmdr1, pfatpase6 and K13-propeller polymorphisms in Luanda, Angola. Malar J. 2015;14:504 10.1186/s12936-015-1018-3 26670642 PMC4681156

[32] Happi CT, Gbotosho GO, Folarin OA, Akinboye DO, Yusuf BO, Ebong OO, et al Polymorphisms in Plasmodium falciparum dhfr and dhps genes and age related in vivo sulfadoxine-pyrimethamine resistance in malaria-infected patients from Nigeria. Acta Trop. 2005;95:183–93. 16023986 10.1016/j.actatropica.2005.06.015

[33] Holmgren G, Gil JP, Ferreira PM, Veiga MI, Obonyo CO, Björkman A. Amodiaquine resistant Plasmodium falciparum malaria in vivo is associated with selection of pfcrt 76T and pfmdr1 86Y. Infect Genet Evol. 2006;6(4):309–14. 16271310 10.1016/j.meegid.2005.09.001

[34] Nawaz F., Nsobya S. L., Kiggundu M., Joloba M. & Rosenthal P. J. Selection of parasites with diminished drug susceptibility by amodiaquine-containing antimalarial regimens in Uganda. J. Infect. Dis. 2009;200,1650–1657. 10.1086/647988 19905933 PMC2782860

[35] Watkins WM, Mosobo M. Treatment of Plasmodium falciparum malaria with pyrimethamine-sulfadoxine: selective pressure for resistance is a function of long elimination half-life. Trans R Soc Trop Med Hyg. 1993;87(1):75–8. 8465404 10.1016/0035-9203(93)90431-o

[36] Diourté Y, Djimdé A, Doumbo OK, Sagara I, Coulibaly Y, Dicko A, et al Pyrimethamine-sulfadoxine efficacy and selection for mutations in Plasmodium falciparum dihydrofolate reductase and dihydropteroate synthase in Mali. Am J Trop Med Hyg. 1999;60(3):475–8. 10466980 10.4269/ajtmh.1999.60.475

[37] Djimdé AA, Doumbo OK, Traore O, Guindo AB, Kayentao K, Diourte Y, et al Clearance of drug-resistant parasites as a model for protective immunity in Plasmodium falciparum malaria. Am J Trop Med Hyg. 2003;69(5):558–63. 14695097

[38] Ogouyèmi-Hounto A, Tuikue NN, Kinde GD, d’Almeida S, Koussihoude L, Ollo Elvire, et al Prevalence of the molecular marker of Plasmodium falciparum resistance to chloroquine and sulphadoxine/pyrimethamine in Benin seven years after the change of malaria treatment policy. Malar J. 2013;12:147 10.1186/1475-2875-12-147 23634705 PMC3651273

[39] World Health Organization: Defining and validating a measure of parasite resistance to sulfadoxine-pyrimethamine (SP) that would be indicative of the protective efficacy of SP for intermittent preventive treatment in infancy (SP-IPTi); 2009. www.who.int/malaria/…/who_sp_ipti_resistance_march_2010.pdf

