# Supplementary material for: Seasonal Malaria Chemoprevention with Sulphadoxine-Pyrimethamine and Amodiaquine Selects Pfdhfr-dhps Quintuple Mutant Genotype in Mali
Source: PLoS One. 2016 Sep 23;11(9):e0162718. doi: 10.1371/journal.pone.0162718 (PMC5035027; doi:10.1371/journal.pone.0162718)
Supplement: S1 File — (PDF) [file pone.0162718.s001.pdf]

ID	Date	InitialVolontary	Sex	FeverHistory	Temprature	Hemoglobin	RDTResultat	*P.falciparum (Trophozoites/microliter)*
1020101	05aug2012	BC	F	1	37.3	7.7	1	104400
1020201	05aug2012	YC	M	1	36.2	10.8	1	60720
1020202	05aug2012	SC	M	0	36.7	10.9		0
1020301	05aug2012	DC	F	1	36.2	11.1		0
1030101	05aug2012	AC	M	1	36.4	9.5	1	0
1030102	05aug2012	BC	M	1	37.1	10		0
1040101	05aug2012	NC	M	0	37.0	11.3		0
1050101	05aug2012	YC	M	0	37.1	12.6		0
6010101	06aug2012	AC	F	1	36.0	9.7		0
6010102	06aug2012	KC	F	1	37.8	6.6	1	29680
6010201	06aug2012	AC	F	1	36.0	8.4		14560
6020101	06aug2012	FC	M	1	36.2	12.3		0
6020102	06aug2012	SC	F	0	36.8	10.6		0
6020201	06aug2012	SC	F	0	36.4	9.9		0
6020202	06aug2012	OC	F	1	36.0	8.3		18120
6020301	06aug2012	DC	M	0	36.2	10.9		0
6030101	06aug2012	SC	F	0	36.2	8.7		440
6030201	06aug2012	KC	F	0	36.1	12.2		0
6030202	06aug2012	SC	F	1	36.7	9.7		0
6030301	06aug2012	SC	F	0	36.5	9.4		62400
6030401	06aug2012	SC	M	0	36.1	9.7		0
6040101	06aug2012	FC	F	0	36.7	12.1		53800
6040201	06aug2012	MC		0	36.2	11.8		0
6040301	06aug2012	SC	M	0	36.0	8.1		7640
6050101	06aug2012	OC	F	1	38.4	5.3	1	13400
6050201	06aug2012	YC	M	0	36.8	10.5		0
6050301	06aug2012	BC	M	0	36.7	11.7		0
6050302	06aug2012	OC	M	0	36.3	12.5		0
6060101	06aug2012	FC	F	1	36.4	10.4		0
6060102	06aug2012	NC	F	0	36.7	10.6		0
6060201	06aug2012	RC	F	0	36.8	9.5		34560
6070101	06aug2012	MC	F	1	37.2	8.5	0	0

6070201	06aug2012	BC	F	0	36.2	11.7		0
6070202	06aug2012	FC	F	0	36.4	10.5		0
6070203	06aug2012	KC	F	1	36.3	10.8		0
6070301	06aug2012	IC	M	0	36.0	10.8		0
6070302	06aug2012	SC	M	0	36.1	10.6		0
6070401	06aug2012	FC	F	0	36.0	10.1		0
6080101	06aug2012	DN	F	1	37.6	9.4	0	0
6090101	06aug2012	SC	F	1	36.0	8.1		0
8010101	08aug2012	SD	M	1	36.7	11.6	1	80
8010102	08aug2012	MD	F	1	36.0	9.3	1	2840
8010201	08aug2012	KD	F	1	36.3	11.3		4000
8020101	08aug2012	SD	M	1	37.2	12	1	920
8020102	08aug2012	ID	M	1	36.0	7.2		0
8020201	08aug2012	MD	M	1	36.0	6.7		0
8030101	08aug2012	MD	F	1	38.4	7.7	1	94720
8040101	08aug2012	RD	F	1	36.3	7.6		0
8040201	08aug2012	KD	F	1	36.0	12.4		5280
8040301	08aug2012	MD	M	1	36.3	9.5	1	200
8040302	08aug2012	BD	F	1	37.5	8.5	1	13960
8040401	08aug2012	TD	M	1	37.0	10.2		0
8040501	08aug2012	AD	M	1	36.6	11.5		0
8040601	08aug2012	MD	M	0	36.7	11.2		0
8040602	08aug2012	CD	M	0	36.1	8.4		24160
8040603	08aug2012	TD	M	0	36.4	12.8		0
8040604	08aug2012	ID	M	1	37.0	8.4	1	8640
8040701	08aug2012	SD	F	0	36.0	12		160
8040801	08aug2012	OD	M	0	36.8	10.5		2720
8040901	08aug2012	AD	F	1	36.0	7.3	1	3440
8041001	08aug2012	LD	F	1	36.4	11.7		720
8041101	08aug2012	TD	F	1	36.0	10.6	1	120
8050101	08aug2012	KD	M	1	36.0	10.6	0	40480
8050201	08aug2012	SD	M	1	36.0	7.6		0
8060101	08aug2012	HS	M	1	36.0	8.8	1	0
8060201	08aug2012	YS	M	0	36.0	10.4		560

8060202	08aug2012	FS	F	0	36.0			6080
8070101	08aug2012	OD	M	0	37.1	11.3		1040
8070102	08aug2012	AD	F	1	37.3	11.2	1	5920
8080101	08aug2012	AD	F	1	36.7	10.7	1	3320
8080102	08aug2012	AD	M	0	36.8	11.5		680
8080201	08aug2012	SD	F	1	36.4	10.3	1	6840
8080301	08aug2012	OD	M	1	36.7	8.6	1	0
1010101	05aug2012	AC	M	0	36.2	8.4		0
1010102	05aug2012	MC	M	1	36.7	8.1	1	0
1010201	05aug2012		F	0	36.8	9.3		0
1010301	05aug2012	FC	M	1	37.7	10.1	1	69640
1020102	05aug2012	IC	M	0	36.7	10.7		0
1020302	05aug2012	SC	F	0	36.5	10.5		0
1060101	05aug2012	AC	M	0	36.4	11.3		0
1060102	05aug2012	MC	F	0	36.1	10.7		0
1070101	05aug2012	MC	F	1	36.1	10.9	1	0
1070201	05aug2012	LC	M	1	36.0	11.2		0
1070202	05aug2012	LC	M	0	36.7	10.4		280
1080101	05aug2012	SC	M	1	35.9	9.2		0
1080102	05aug2012	OC	M	0	35.5	11.1		1720
1080201	05aug2012	DC	M	0	36.0	9.2		0
1080202	05aug2012	DC	M	0	35.2	10.7		0
1080301	05aug2012	AC	F	0	36.1	10.3		0
1080501	05aug2012	TC	M	0	36.7	9.8		3920
1090101	06aug2012	OC	M	1	36.1	10.3	0	0
1090201	06aug2012	IC	M	1	37.3	6.1	1	0
1100101	06aug2012	KC	F	1	37.9	9.5	1	117680
1100201	06aug2012	SC	M	1	36.6	8.8	1	17800
11010101	05aug2012	CG	M	1	36.5	10.6		0
11010201	05aug2012	ND	F	0	36.4	11.7		0
11010301	05aug2012	SD	M	1	37.0	6.1		0
11010401	05aug2012	DD	F	0	36.7	10.7		0
11010501	05aug2012	FD	F	1	36.7	11	0	0
11010601	05aug2012	KD	F	0	35.7	11.8		200

11010701	05aug2012	CD	F	0	35.7	8.2		160
11010801	05aug2012	BD	F	0	37.7	8.1	1	240
11010901	05aug2012	CD	F	0	36.2	9.1		0
11011001	05aug2012	MD	F	0	36.2	9.1		0
11011101	05aug2012	JD	F	1	39.8	6.5	1	100400
11011102	05aug2012	SD	F	1	35.6	10.3		0
11011201	05aug2012	MD	F	0	37.0	11.4		0
11011202	05aug2012	SD	M	0	36.8	11.6		0
11011203	05aug2012	SD	M	0	36.4	8.7		0
11011301	05aug2012	CD	F	0	36.1	10		0
11020101	05aug2012	BD	F	1	36.7	7.8	1	79640
11020201	05aug2012	CS	F	0	35.9	10.7		0
11020301	05aug2012	SK	M	0	37.3	9.7		0
11020401	05aug2012	DK	F	0	36.2	12.7		0
11020501	05aug2012	SK	M	0	37.4	12.7	0	0
11030101	05aug2012	DD	M	1	37.1	10.8	0	0
11030201	05aug2012	ED	M	0	36.8	5.6		0
11040101	05aug2012	DD	M	0	36.3	9.7		0
11040201	05aug2012	SD	M	0	36.4	10.1		0
11040301	05aug2012	ZD	M	0	36.6	11.5		5200
11040302	05aug2012	DD	M	0	36.4	9.9		0
11050101	05aug2012	BK	M	0	37.1	10.1		0
11060101	05aug2012	AD	F	1	36.1	10.7	1	157520
11060102	05aug2012	MS	M	0	36.2	11.2		0
11060201	05aug2012	GD	F	0	35.9	8		0
11070101	05aug2012	ID	M	0	36.5	9.9		0
11070201	05aug2012	OD	M	0	36.1	11.3		0
1110101	06aug2012	AC	F	1	37.8	7.8	1	0
1120101	06aug2012	OC	M	1	36.9	8.5	1	0
1120201	06aug2012	SC	M	1	36.1	7	1	3120
1130101	06aug2012	BC	F	1	36.0	11.5	0	0
1130102	06aug2012	DC	F	1	36.7	12.5	0	0
1130201	06aug2012	SC	M	1	36.9	11.1	0	0
12010101	06aug2012	HD	F	1	36.3	10.1	1	0

12020101	06aug2012	RD	F	1	37.6	9.1	1	37960
12020102	06aug2012	HD		1	36.7	12.9	1	0
12020201	06aug2012	SD	M	1	36.9	10.9	1	720
12020202	06aug2012	SD	M	0	36.4	10.3		0
12020301	06aug2012	SD	M	1	36.2	7.7	1	1880
12020302	06aug2012	OD	M	1	36.4	9.2		
12020401	06aug2012	SD	M	0	36.7	11.3		120
12020402	06aug2012	KD	F	1	37.8	9	1	80
12020403	06aug2012	KD	F	0	36.6	9.7		0
12020501	06aug2012	YD	M	0	35.9	9.5		0
12020601	06aug2012	SD	F	0	36.5	13		0
12020701	06aug2012	YD	M	0	37.0	11.2		0
12020801	06aug2012	MD	M	0	36.2	11.8		0
12020901	06aug2012	BD	F	0	37.0	9.6		0
12020902	06aug2012	MD	M	0	36.7	9		0
12021001	06aug2012	MD	F	1	36.2	9.9		0
12021101	06aug2012	ID	M	0	35.6	10.3		0
12021201	06aug2012	GD		0	35.9	11.3		0
12021301	06aug2012	KD	F	0	36.3	11.1		0
12030101	06aug2012	DC	F	0	35.9	10.3		0
12030102	06aug2012	SC	M	0	35.7	10.9		0
12030201	06aug2012	SC	M	0	36.4	10.5		0
12030202	05aug2012	DC	F	1	40.2	7.7	1	56280
12040101	06aug2012	SD	F	0	36.7	10.4		1040
12040201	06aug2012	MD	M	0	35.9	8.3		0
12050101	06aug2012	HD	M	0	35.9	6		0
12050102	06aug2012	KD	F	0	36.0	10.9		0
12050201	06aug2012	KD	F	0	37.4	9.8		0
12050202	06aug2012	SD	F	1	35.8	6.7	1	1680
12050301	06aug2012	BD	F	1	37.7	10.5	1	3840
12050302	06aug2012	BD	F	0	36.3	8.5	1	0
12050401	06aug2012	SD	F	1	36.2	7.3	1	0
12050501	06aug2012		F	0	36.4	8.9		1920
13010101	08aug2012	AK	M	0	36.4	9.8		0

13010102	08aug2012	MK	M	0	36.9	11.9		0
13020101	08aug2012	MK	M	0	35.7	13.2		0
13020201	08aug2012	MK	M	0	36.2	9.2		0
13020301	08aug2012	SK	M	0	37.2	10.4		0
13020302	08aug2012	SK	F	0	36.0	11.3		0
13030101	08aug2012	AK	F	0	36.6	11.7		0
13040101	08aug2012	YK	M	0	36.1	11.1		0
13040201	08aug2012	DK	F	0	36.1	9.3		0
13040302	08aug2012	AK	M	0	35.8	11.6	1	0
13050101	08aug2012	AK	F	0	35.9	12.2		0
13050102	08aug2012	MK	M	1	35.9	11	1	0
13060101	08aug2012	AK	F	0	36.7	13		0
13060102	08aug2012	NK	F	0	37.0	12.4	1	0
13060201	08aug2012	SK	M	0	35.6	11		0
13060301	08aug2012	DK	M	0	37.0	11	0	0
13060401	08aug2012	SK	F	0	36.4	10.3		0
13060402	08aug2012	MK	M	0	35.9	10.2		0
13060501	08aug2012	AK	F	0	36.7	11.1		0
13060601	08aug2012	SK	M	0	36.8	10		0
13060701	08aug2012	SK	M	1	36.8	7.5		0
13060702	08aug2012	DK	M	0	36.4	13.5		0
13070101	08aug2012	DT	F	0	36.1	12.2		0
13070102	08aug2012	AK	F	0	35.3	13	0	0
13080101	08aug2012	DK	F	0	35.9	12.2		0
13080201	08aug2012	SK	F	0	36.2	9.7		0
13080202	08aug2012	RK	F	0	36.8	10.9		0
13080301	08aug2012	MK	M	0	36.2	12.7		0
13080302	08aug2012	AK	M	0	36.1	12.5		0
13080401	08aug2012	DK	M	0	36.8	11.2		0
13080402	08aug2012	TK	M	0	36.1	8.9		0
13080501	08aug2012	SK	M	1	37.0	10	0	80
17010101	09aug2012	AK	F	1	37.4	12	0	0
17010201	09aug2012	MK	F	0	36.0	12.1		0
17010202	09aug2012	MK	M	0	36.7	9.4		0

17010301	09aug2012	RC	M	0	36.2	12.2		0
17010302	09aug2012	KC	F	0	36.1	10.2		0
17010401	09aug2012	AD	F	0	36.1	12.2		0
17010402	09aug2012	SD	M	0	36.2	10.2		0
17010501	09aug2012	BD	F	0	36.4	10.7		0
17010601	09aug2012	OD	M	0	36.0	13.3		0
17010701	09aug2012	AK	F	0	35.9	9.8		0
17010702	09aug2012	BK	F	0	37.1	10.9		0
17010801	09aug2012	NC	F	0	35.9	10.9		0
17020101	09aug2012	IT	M	0	35.3	9.5		0
17020201	09aug2012	SN	F	1	36.2	12.4	0	0
17030101	09aug2012	DG	M	1	36.4	9.9	0	0
17030102	09aug2012	MG	F	0	37.0	11.7		0
17030201	09aug2012	YG	M	0	36.9	10.5		0
17030401	09aug2012	DB	F	0	37.1	12.5		0
17030501	09aug2012	KT	F	0	37.5	13.6	0	0
17040101	09aug2012	ID	M	0	36.1	10.5		0
17040201	09aug2012	DD		0	35.6	12		0
17040301	09aug2012	DK	M	0	36.8	9.9		0
17050101	09aug2012	BT	M	0	36.7	11.5		0
17050102	09aug2012	AT		0	35.6	11.3		0
17060101	09aug2012	SD	F	0	36.3	12.5		0
17070101	09aug2012	BB	F	1	36.3	11.2	0	0
17070102	09aug2012	KB	F	0	36.4	11		0
17070201	09aug2012	SK	M	1	36.7	9.9	0	0
17070202	09aug2012	AK	F	1	37.3	10	1	46800
17070301	09aug2012	MK	F	0	36.5	10		0
17070302	09aug2012	SK	M	0	36.1	12.5		0
17070303	09aug2012	BK	M	0	36.3	10.8		0
17070401	09aug2012	SS	M	0	37.1	11		0
17070402	09aug2012	BS	F	1	36.9	10.1		0
17070403	09aug2012	OS	M	0	36.7	11.4		0
17070501	09aug2012	SD	M	0	36.2	10.6		0
17070601	09aug2012	MK	F	0	36.2	11.3		0

19010101	09aug2012	DC	F	0	36.2	11.4		0
19010102	09aug2012	BC	M	0	36.7	9.8		0
19010201	09aug2012	DD	F	0	35.9	10.2		0
19020101	09aug2012	TC	F	0	35.6	8.9		0
19020201	09aug2012	MC	M	0	36.6	9		0
19020301	09aug2012	KC	F	0	36.2	9		0
19030101	09aug2012	BB	M	0	37.0	9.2		0
19030102	09aug2012	AB	F	0	36.0	10.4		0
19030201	09aug2012	MB	M	0	36.7	11		0
19030202	09aug2012	RB	F	0	36.1	9.7		0
19030301	09aug2012	HB	F	0	35.6	9.9		0
19030401	09aug2012	AS	M	0	36.9	12.4		0
19030402	09aug2012	LS	F	0	36.8	10.1		0
19040101	09aug2012	BD	M	0	36.4	10.5		0
19040201	09aug2012	KK	F	0	36.8	12.9		0
19040301	09aug2012	AK	F	0	36.5	10.6		0
19050101	09aug2012	AD	F	0	36.4	11.6		0
19050102	09aug2012	SD	M	0	36.8	10.7		0
19050201	09aug2012	AT	M	0	36.8	7.6		0
19050301	09aug2012	MM	M	0	36.1	12.8		0
19060101	09aug2012	HD	M	0	36.9	10.9		0
19060102	09aug2012	GD	M	0	37.1	11.5	0	0
19070101	09aug2012	AC	M	0	36.8	11.8		320
19080101	09aug2012	BT	F	0	36.4	12		0
19080201	09aug2012	BT	M	0	36.1	11.7		0
19080301	09aug2012	MS	M	0	36.4	9.6		0
19090101	09aug2012	MD	M	0	35.8	10.9		0
19090102	09aug2012	ZD	F	0	35.9	11.5		0
19100101	09aug2012	SD	F	0	36.4	10.4		0
19110101	09aug2012	BK	M	0	36.4	10.9		0
19120101	09aug2012	KT	F	1	37.1	9.4	0	0
19120201	09aug2012	SD	F	0	36.2	6.8		0
21010101	05aug2012	MD	F	0	35.9	12.3		0
21010102	05aug2012	SD	F	0	35.9	10.4		0

21010201	05aug2012	LD	M	0	36.1	9.4		0
21010301	05aug2012	BD	M	0	36.4	11.1		0
21010401	05aug2012	DD	M	0	38.2	11.1	0	0
21010501	05aug2012	KD	F	0	36.6	10.9		0
21010502	05aug2012	OD	F	0	36.4	8.7		0
21020101	05aug2012	RD	F	1	38.7	6.8	1	1080
21020102	05aug2012	FD	F	1	36.1	10.3		0
21030101	05aug2012	KD	F	0	35.6	11.8		0
21030201	05aug2012	RD	F	0	36.4	12.7		0
21030301	05aug2012	ZD	M	1	36.1	10.4	1	640
21030302	05aug2012	KD	M	0	36.2	10.6		0
21040101	05aug2012	SK	F	1	36.3	6.1		0
21050101	05aug2012	KC	M	1	38.8	11.1	1	1660000
21050102	05aug2012	AC	M	1	37.7	9.4	0	0
21050201	05aug2012	AD	M	1	36.6	10.9		0
21050301	05aug2012	YC	M	0	36.3	10.9		0
21050302	05aug2012	SC	F	1	36.4	9.5	0	0
21050401	05aug2012	ID	M	0	36.6	8.9		0
21050501	05aug2012	MD	F	0	36.1	11.7		0
21050502	05aug2012	CD	F	0	35.7	10.3		0
21050601	05aug2012	DC	F	0	36.2	12.9		0
21060101	05aug2012	AS	M	0	36.6	10		720
21060201	05aug2012	CS	M	0	36.2	8.7		0
21060301	05aug2012	BT	M	0	36.2	9.6		0
21060302	05aug2012	BT	F	0	36.4	10.4		0
21060401	05aug2012	AS	F	0	36.4	10		160
21060501	05aug2012	AS	M	0	36.1	9.7		0
21060502	05aug2012	AS	F	0	36.1	9.7		0
21060601	05aug2012	AS	M	0	36.2	8.3		0
21060701	05aug2012	SD	M	1	36.2	7.3		120
22010101	04aug2012	MK	F	0	36.2	10		0
22010102	04aug2012	SK	M	0	36.9	10.5		0
22020101	04aug2012	DD	F	1	35.1	9.1	0	0
22020201	04aug2012	DD	M	0	36.2	10.5		0

22030101	04aug2012	NC	M	1	36.8	10.4	1	0
22030201	04aug2012	OT	F	0	36.6	9.7		0
22030301	04aug2012	FC	F	0	36.2	10.3		0
22030401	04aug2012	MC	M	1	36.8	10.5	0	0
22030501	04aug2012	BC	M	0	36.7	11.2		0
22030601	04aug2012	DC	F	0	37.0	10.9		0
22030701	04aug2012	AC	F	1	36.1	10.4		0
22040101	04aug2012	FC	F	0	36.4	11.1		0
22040201	04aug2012	MC	F	0	37.0	9.3		0
22040301	04aug2012	NC	M	0	36.8	10.7		0
22040302	04aug2012	BD	M	1	37.1	10.7	0	0
22040401	04aug2012	SC	F	0	35.7	11.2		0
22050101	04aug2012	AC	F	0	35.6	10.7		0
22050201	04aug2012	SC	F	0	36.4	12.4		0
22050301	04aug2012	AC	F	0	36.7	10.6		0
22050302	04aug2012	BC	F	1	36.1	11		0
22060101	04aug2012	MS	M	0	37.0	8.8		0
22060201	04aug2012	BT	M	0	36.1	8.4		240
22060202	04aug2012	LT	M	1	36.4	10.6		0
22060301	04aug2012	NS	F	0	36.4	8.4		2040
22060401	04aug2012	AT	M	1	37.7	7.6	1	12800
22060501	04aug2012	SD	M	0	36.4	12		160
22060502	04aug2012	MD	F	0	36.4	11.5		0
22060601	04aug2012	AT	F	1	36.4	11.1		0
22060602	04aug2012	KT	F	0	36.7	10.6		0
22070101	04aug2012	AC	M	0	35.9	9.9		0
22070201	04aug2012	FT	F	1	36.7	10.9		0
22070301	04aug2012	DC	F	1	37.6	10.8	0	0
22070302	04aug2012	CC	F	0	36.8	12.9		0
22070401	04aug2012	BC	F	0	36.2	12.4		0
23010101	06aug2012	NC	F	0	36.0	11		0
23010102	06aug2012	SC	M	1	35.6	8.8		0
23010201	06aug2012	UC	M	0	36.0	10.4		4680
23010202	06aug2012	JC	F	1	37.7	8.4	0	0

23010301	06aug2012	RC	F	0	35.8	9.7		0
23020101	06aug2012	SC	F	0	36.6	12		0
23020102	06aug2012	IC	M	0	36.4	9.7		0
23020201	06aug2012	FC	F	0	35.6	10		0
23020301	06aug2012	SC	F	0	36.1	6.6		0
23020302	06aug2012	BC	F	0	36.0	10.4		0
23020401	06aug2012	AC	M	0	36.4	12.6		0
23020501	06aug2012	BC	F	0	36.4	8.3		0
23020601	06aug2012	TD	F	1	35.6	9.8		0
23020602	06aug2012	MD	M	0	35.7	9.7		0
23020701	06aug2012	AC	M	0	36.2	11.3		0
23020702	06aug2012	DC	F	0	35.6	10.1		0
23030101	06aug2012	AC	M	0	36.0	10.3		0
23040101	06aug2012	FC	F	1	37.0	6.3	1	1480
23050101	06aug2012	KC	M	0	36.4	11.6		0
23050102	06aug2012	AC	M	0	36.2	10.1		0
23050103	06aug2012	SC	M	0	37.7	10.4	0	0
23050201	06aug2012	ST	F	1	38.8	8.3	1	45480
23060101	06aug2012	SC	M	0	35.9	10.5		0
23060102	06aug2012	DC	M	0	35.6	8.7		0
23060201	06aug2012	AC	M	0	35.9	9.5		0
23060202	06aug2012	AC	F	0	35.6	10.5		0
23060301	06aug2012	ND	F	0	36.4	11.6		0
23060302	06aug2012	BS	F	0	35.2	11.1		0
23070101	06aug2012	KD	F	1	36.7	11.3	0	0
23070102	06aug2012	SD	F	0	36.4	10.7		0
23070201	06aug2012	KD	M	0	36.5	10		1400
23070202	06aug2012	BD	M	0	36.1	8.2		0
26010101	06aug2012	BT	M	0	36.7	10.2		12520
26010201	06aug2012	TK	F	0	35.6	7.2	1	178840
26010202	06aug2012	AK	F	0	37.3	4.7	1	0
26010301	06aug2012	BK	F	0	35.8	10		880
26020101	06aug2012	SC	F	0	35.9	11.2		0
26020201	06aug2012	TB	F	0	36.2	9.9		0

26020301	06aug2012	KC	F	0	35.6	10.3		1840
26020401	06aug2012	DC	F	0	36.2	10.3		0
26020402	06aug2012	BC	M	0	36.4	7.3		0
26030101	06aug2012	IB	M	0	37.0	9.6	1	0
26030102	06aug2012	MB	F	0	36.4	8.8		0
26030201	06aug2012	AB	M	0	36.9	10		520
26030301	06aug2012	DB	M	0	36.1	10.9		1200
26030302	06aug2012	MB	F	0	36.2	9.9		0
26030401	06aug2012	SB	M	0	36.7	8.4		1080
26030501	06aug2012	YB	M	0	37.3	9.7	1	5920
26040101	06aug2012	MB	F	0	36.7	10.2		840
26040102	06aug2012	BB	F	0	38.4	5.9	1	30480
26040201	06aug2012	RB	F	0	36.7	11.7		400
26040202	06aug2012	AB	F	0	36.1	9.1		15920
26040301	06aug2012	RB	F	0	35.7	9.9		80
26040401	06aug2012	GK	M	0	35.9	8.5		0
26040501	06aug2012	LB	M	0	36.8	9.1		120
26040601	06aug2012	AB	M	0	36.8	9.9		0
26050101	06aug2012	TB	M	0	36.7	7.2		560
26050201	06aug2012	1	M	1	37.0	12.2	1	920
26050301	06aug2012	SB	F	0	36.1	12.1		320
26050401	06aug2012	DB	F	1	37.1	11.2	1	33880
26050501	06aug2012	AB	F	0	36.4	8.8		1040
26050601	05aug2012	YB	M	0	36.7	9.8		0
26060101	06aug2012	AT	F	0	36.2	9.6		1200
26060201	06aug2012	ST	F	0	36.4	9.4		240
26060301	06aug2012	AT	M	1	37.7	6.5	1	3960
27010101	05aug2012	OK	M	1	36.0	10.4		2760
27020101	05aug2012	KD	F	0	36.8	12		0
27020102	05aug2012	ZM	M	1	36.8	9.6		0
27020201	05aug2012	AM	F	0	36.7	10.6		0
27020202	05aug2012	KM	F	1	36.5	9.1		0
27030101	05aug2012	AM	M	0	36.1	11.6		0
27040101	05aug2012	YT	M	0	36.4	10.4		0

27040201	05aug2012	AT	F	0	36.4	11.8		0
27040202	05aug2012	MT	F	0	37.0	11		0
27040301	05aug2012	AK	F	0	36.0	11.6		0
27040302	05aug2012	KK	F	0	36.0	9.8		0
27050101	05aug2012	KM	M	0	36.2	10.3		0
27050201	05aug2012	NM	F	1	37.4	5		25680
27060101	05aug2012	KT	M	0	36.0	10.3		200
27060201	05aug2012	ST	M	1	38.1	9.2	1	1360
27060202	05aug2012	RT	F	0	36.0	9.4		0
27060301	05aug2012	FT	F	1	36.4	6		0
27060401	05aug2012	MS	M	0	37.1	11		0
27060501	05aug2012	MK	F	0	36.1	11.5		0
27060601	05aug2012	AT	M	1	36.0	10.6		0
27070101	05aug2012	AS		1	37.0	9.2		0
27070102	05aug2012	MS	F	1	38.8	5.2	1	0
27080101	05aug2012	AD	F	1	37.4	7.9	1	160
27080102	05aug2012	ND	M	0	36.7	9.7		0
27080201	05aug2012	AD	F	1	36.8	10.9		600
27080202	05aug2012	SD	M	0	36.0	7.9		0
27080301	05aug2012	BD	F	0	36.2	11.2		0
27080401	05aug2012	BD	F	0	36.2	11.7		0
27080501	05aug2012	CD	M	1	36.4	9.4		0
27080502	05aug2012	ED	F	1	37.0	7.5		0
27080503	05aug2012	AD	M	0	36.2	12		0
27090101	05aug2012	YM	F	1	37.1	9	1	120
29010101	05aug2012	KM	F	0	36.2	10.9		0
29020101	05aug2012	AC	M	0	36.7	9.9		0
29020102	05aug2012	SC	M	0	36.2	9.9		0
29020201	05aug2012	IC	M	0	36.4	11.2		0
29020301	05aug2012	SC	M	0	37.1	9.7		0
29020401	05aug2012	DM	F	0	36.0	9.5		0
29030101	05aug2012	YK	M	0	36.7	6.8		0
29030102	05aug2012	LK	F	0	36.0	10.1		0
29030201	05aug2012	YD	M	0	35.6	10.9		0

29040101	05aug2012	DD	M	0	36.2	9.1		440
29050101	05aug2012	RD	F	0	36.0	9.8		720
29050102	05aug2012	FD	M	0	36.4	10.1		0
29050103	05aug2012	LD		0	36.1	10.7		0
29050104	05aug2012	SD	F	0	36.4	9.2		760
29050105	05aug2012	BD	M	0	36.7	10.1		0
29050201	05aug2012	DD	M	0	37.0	9.9		13360
29050202	05aug2012	KD	M	0	35.6	9.7		960
29050203	05aug2012	AD	M	0	36.0	10.6		0
29050301	05aug2012	AD	F	1	38.2	8.8	1	27920
29050401	05aug2012	AC	F	1	36.7	8		10600
29050402	05aug2012	SC	M	1	36.4	8		93520
29050403	05aug2012	AC	F	0	36.7	10.6		0
29050501	05aug2012	ND	F	0	36.0	8.8		880
29050502	05aug2012	TD	F	0	37.0	12		520
29050503	05aug2012	MD	F	0	36.1	9.7		0
29060101	05aug2012	AK	M	0	38.6	10.2	1	22240
29060102	05aug2012	MK	M	0	36.0	9.5		640
29070101	05aug2012	NM	M	0	36.2	9.3		0
29080101	05aug2012	RK	F	0	36.7	9.9		10400
29080102	05aug2012	YK	M	0	36.6	9.1		0
29080201	05aug2012	SK	M	1	36.4	5.4	1	2320
29080202	05aug2012	OK	M	0	36.0	11.6		0
33010101	05aug2012	MK	F	0	36.2	10.9		0
33010201	05aug2012	FK	F	0	36.5	12.6		1640
33010301	05aug2012	DK	M	0	35.8	11.1		0
33010401	05aug2012	SK	F	1	37.1	12.6	1	1680
33010402	05aug2012	FK	M	0	36.7	10.1		200
33020101	05aug2012	ST	M	1	35.9	9.6	1	29680
33020102	05aug2012	AT	M	0	37.0	10		0
33030101	05aug2012	MC	M	0	36.2	12.3		10160
33030201	05aug2012	BT	M	0	36.8	11.8		30600
33030301	05aug2012	FT	F	0	36.6	12		5880
33040101	05aug2012	NC	F	1	36.0	9.8	1	0

33040102	05aug2012	SC	F	0	35.9	11.1		0
33050101	05aug2012	MC	F	1	36.6	10	1	400
33050201	05aug2012	YC	M	1	37.0	12		0
33050202	05aug2012	AT	F	0	36.3	11.5		0
33060101	05aug2012	TC	M	1	37.3	7.4	1	10000
33070101	05aug2012	ST	M	1	36.7	11.9		0
33080101	05aug2012	MT	M	0	36.3	11.7		0
33080201	05aug2012	CT	F	1	36.4	11.7	1	200
33090102	05aug2012	ST	M	0	36.8	9.7		0
33090201	05aug2012	DT	F	1	36.7	11.9		0
33100101	05aug2012	MT	M	1	37.3	10.3	1	2560
33100102	05aug2012	NT	F	0	36.8	10.4		0
33100201	05aug2012	AT	F	1	35.6	10.9	1	1200
33100301	05aug2012	MT	F	1	37.7	11.6	1	800
33110101	05aug2012	ST	M	0	35.6	11		1640
33110102	05aug2012	TT	F	1	36.6	9.9	0	0
33120101	05aug2012	MT	F	1	36.4	7.9	1	3000
33120201	05aug2012	AT	M	0	36.7	10.8		0
33130101	05aug2012	BT	F	0	37.8	11.2	1	3760
33140101	05aug2012	ST	M	1	36.4	10.6		0
33150101	05aug2012	AD	F	0	36.4	11.2		0
34010101	09aug2012	FD	F	0	36.6	11.8		0
34010201	09aug2012	KD	F	0	35.9	11.9		0
34010202	09aug2012	FD	F	0	35.6	9.3		0
34010301	09aug2012	BT	M	1	37.8	11.9	0	0
34010302	09aug2012	MT	F	0	36.8	10.3		0
34010401	09aug2012	FS	F	0	36.1	12.2		0
34010501	09aug2012	LT		0	37.0	10.7		0
34020101	09aug2012	DS	M	1	37.1	9.8	0	0
34030101	09aug2012	MT	F	0	35.3	9.9		0
34030201	09aug2012	MW	M	0	36.9	10.5		0
34040101	09aug2012	MC	M	0	36.5	10.2		0
34040102	09aug2012	KC	F	0	36.8	8.3		0
34040201	09aug2012	MC	M	0	37.0	10		0

34040301	09aug2012	LK	M	0	36.4	10.2		0
34050101	09aug2012	ZA	F	1	37.4	11	0	0
34050201	09aug2012	FD	F	0	37.0	13.5		0
34060101	09aug2012	AT	F	0	36.7	12		0
34060201	09aug2012	CC	M	0	36.8	11.7		0
34060301	09aug2012	SS	F	0	36.7	9.6		0
34060302	09aug2012	IS	M	1	36.3	11.3	0	0
34070101	09aug2012	HS	F	0	35.5	11.1		0
34070201	09aug2012	CS	M	1	35.6	11.6	0	0
34070301	09aug2012	AT	M	0	36.7	10.8		0
34070302	09aug2012	CT	F	0	36.7	10.2		0
34070401	09aug2012	AS	M	0	36.4	11.7		0
34070501	09aug2012	AS	M	0	36.4	11.4		0
34070502	09aug2012	AS	F	0	36.8	10.9		0
34080101	09aug2012	SK	M	0	36.2	11.9		0
34080201	09aug2012	SS	F	1	36.9	11.3		120
34090101	09aug2012	KB	M	1	36.5	9.4		0
34090102	09aug2012	MB	M	0	36.3	12.1		0
34090201	09aug2012	MK	F	0	36.6	8.8		0
34090301	09aug2012	EK	F	1	36.8	10		0
34100201	09aug2012	BB	M	0	37.6	9.3		0
36010101	07aug2012	ID	M	1	35.6	11.4	1	2960
36010102	07aug2012	YD	M	1	35.9	12.5	0	0
36010201	07aug2012	SD	F	1	35.6	5	1	440
36010202	07aug2012	SD	M	0	35.9	10.1		0
36010301	07aug2012	CD	M	0	36.8	11.3		0
36020101	07aug2012	FB	F	1	36.4	8.5	1	3040
36030101	07aug2012	DK	M	1	35.9	9.8	0	0
36030201	07aug2012	FK	F	0	36.4	11		0
36030301	07aug2012	MK	M	0	36.4	9.6		0
36030401	07aug2012	AK	M	0	36.5	12.4		0
36040101	07aug2012	MT	F	1	35.4	10.5	0	0
36040102	07aug2012	DT	F	0	36.2	10		0
36040201	07aug2012	ST	M	1	36.7	8.5	1	80

36050101	07aug2012	MD	M	1	37.3	10.9	1	0
36050102	07aug2012	SD	F	0	35.8	11.8		0
36050201	07aug2012	MT	M	1	36.1	10.1	0	0
36050301	07aug2012	DT	M	1	35.6	10.4	1	0
36060101	07aug2012	HT	F	1	38.2	10.4	1	6520
36060102	07aug2012	DT	M	1	36.9	9.8	0	0
36070101	07aug2012	MD	M	0	35.0	10.3		0
36080101	07aug2012	FS	F	0	35.9	12.1		0
36080102	07aug2012	BS	M	0	35.7	9.3		520
36090101	07aug2012	LD	M	0	35.9	12.6		0
36100101	07aug2012	MD	M	0	36.1	12.1		0
36100102	07aug2012	MD	F	0	35.9	11		0
36110101	07aug2012	SD	F	0	35.6	12.5		0
36120101	07aug2012	SD	M	0	35.6	12.5		0
36130101	07aug2012	MS	F	0	35.0	12.3		0
36140101	07aug2012	MD	F	1	35.9	10.3	0	0
36150101	07aug2012	MT	F	0	36.7	8.5		200
36150102	07aug2012	KT	F	0	35.3	11		0
36160101	07aug2012	KT	M	1	35.3	12.6	0	0
36170101	07aug2012	MD	F	1	36.1	10.3	0	0
36170201	07aug2012	OD	M	1	36.1	9	1	0
38010101	08aug2012	LD	F	1	35.7	7.6	1	440
38010102	08aug2012	MD	F	1	35.7	9.5	1	2080
38020101	08aug2012	TD	M	1	35.9	10.7	1	0
38020201	08aug2012	HD	F	0	36.6	12.3		0
38030101	08aug2012	SD	M	0	36.2	12.6		4160
38040101	08aug2012	MG	F	1	35.9	10.3	0	0
38040102	08aug2012	IG	M	1	36.4	11.3	1	5400
38040201	08aug2012	OG	M	1	36.1	9.9	0	0
38040301	08aug2012	KG	M	0	36.1	11.4		600
38040302	08aug2012	YG	M	0	35.7	9.4		0
38050101	08aug2012	CD	M	0	35.9	8.3		22280
38060101	08aug2012	RD	F	0	35.9	11.6		3120
38060102	08aug2012	AD	M	0	35.9	11.1		0

38060201	08aug2012	MD	M	1	35.9	9.6	1	16840
38060202	08aug2012	RD	F	1	37.9	5.8	1	83400
38060301	08aug2012	OD	M	0	35.7	7.5		0
38070101	08aug2012	MD	M	1	36.7	10.3	1	0
38070102	08aug2012	RD	F	1	36.7	10.3	1	35640
38070201	08aug2012	KD	F	1	35.6	10	1	0
38080101	08aug2012	CD	M	1	36.7	11.5	1	2600
38080201	08aug2012	SD	M	1	35.6	10.3	1	0
38080202	08aug2012	FD	F	1	35.7	9.7	1	5560
38090101	08aug2012	AD	F	0	36.1	12.4		2800
38090102	08aug2012	SD		1	36.1	9.6	1	160
38090103	08aug2012	HD	F	1	35.8	10.2	1	14040
38090201	08aug2012	SD	M	1	35.6	7.3	1	7920
38100101	08aug2012	DG	F	1	35.9	11.9	1	1360
38100102	08aug2012	DG	M	1	35.9	11.4	0	0
38110101	08aug2012	BG	M	1	35.6	8	1	6560
38110201	08aug2012	LG		0	37.1	11		520
38110202	08aug2012	RG	F	0	35.7	9.2		0
38120101	08aug2012	MS	F	1	36.1	10.1	1	7120
38120102	08aug2012	YS	M	1	36.1	12.5	1	2280
38130101	08aug2012	AD	F	1	36.1	11.3	0	0
40010101	07aug2012	AD	F	0	36.5	12.2		0
40010102	07aug2012	AD	F	0	36.0	10		0
40010201	07aug2012	SD	M	1	36.7	8.9		79920
40010202	07aug2012	LD	M	1	36.7	10.5		133160
40010301	07aug2012	AD	M	1	36.7	6.9	1	200
40010302	07aug2012	FD	F	0	36.0	9.4		2320
40010303	07aug2012	MD	M	1	39.0	9.8	1	58880
40010401	07aug2012	SD	M	0	36.0	9.9		0
40010501	07aug2012	KD	M	0	36.4	10.6		0
40010502	07aug2012	OD	M	1	36.0	8.5		0
40010601	07aug2012	MD	M	0	36.1	12.4		2880
40010701	07aug2012	KD	F	0	36.3	11.5		1720
40010801	07aug2012	FD	M	1	36.1	6.6	1	0

40010802	07aug2012	LD	M	1	36.4	7.8	1	2280
40010803	07aug2012	RD	F	0	36.0	12.7		200
40010804	07aug2012	KD	F	0	36.1	9.2		0
40020101	07aug2012	CD	F	0	36.1	9.6		6000
40030101	07aug2012	SD	M	0	36.4	10.7		0
40040101	07aug2012	ED	F	1	38.4	10.4	0	0
40050101	07aug2012	RC	F	1	40.1	7.2	1	150640
40050102	07aug2012	MD	F	0	36.7	11.7		0
40060101	07aug2012	KD	F	1	36.0	5.2		400
40070101	07aug2012	MC	M	0	36.4	9.5		1840
40070102	07aug2012	AC	M	0	37.0	10.4		1120
40080101	07aug2012	SC	F	0	37.0	11.4		0
40090101	07aug2012	LD	F	1	36.1	11	1	1080
40090201	07aug2012	AD	F	0	36.4	10.3		0
40090301	07aug2012	DD	M	0	37.4	6.8	1	960
40090401	07aug2012	MD	F	1	37.0	10.1		0
40090402	07aug2012	SD	M	1	36.8	9		0
40100101	07aug2012	DD	F	1	36.7	8.2		400
40100201	07aug2012	DD	F	0	37.3	9.9		46360
40100301	07aug2012	OD	F	0	36.8	9.5		0
42010101	07aug2012	DS	M	0	36.4	10.2		0
42020101	07aug2012	AS	F	0	35.8	10.9		0
42020201	07aug2012	LS	M	0	36.4	10.3		0
42020202	07aug2012	AS	F	1	38.6	6.9	1	178040
42020301	07aug2012	SS	F	0	37.2	8.9	0	0
42020401	07aug2012	FS	F	0	37.0	6.1	1	18280
42020402	07aug2012	AS	M	1	38.2	4.9	1	13480
42020501	07aug2012	AS	F	0	36.4	9.2		40
42030101	07aug2012	LS	M	0	36.7	11.6		320
42030102	07aug2012	YS	M	0	35.7	9.7		0
42030201	07aug2012	RS	F	0	36.7	10.2		
42030301	07aug2012	LS	M	1	39.3	11.4	0	0
42030302	07aug2012	DS	F	0	36.1	10.6		0
42030401	07aug2012	YS	M	0	36.2	11.4		8320

42030501	07aug2012	DS	M	0	36.4	11		0
42040101	07aug2012	AS	F	0	36.6	6.1		680
42040102	07aug2012	IS	M	0	36.4	8.3		0
42040201	07aug2012	TS	F	0	36.1	10.6		0
42040301	07aug2012	AS	M	0	36.2	8.7		0
42040302	07aug2012	BS	F	0	36.4	8.5		0
42040401	07aug2012	AS	M	0	36.1	12.2		0
42050101	07aug2012	DS	F	0	35.9	11.5		0
42050201	07aug2012	RS	F	0	36.2	11.4		0
42050202	07aug2012	RS	F	0	36.0	8.8		0
42050301	07aug2012	MS	M	0	35.6	10.9		3000
42050401	07aug2012	MS	F	0	36.1	6.8		0
42050402	07aug2012	SS	F	0	35.9	9.1		0
42060101	07aug2012	KT	F	0	36.2	9.8		0
42060201	07aug2012	IS	M	0	38.0	8.6	1	15320
42060202	07aug2012	DS	M	0	36.7	8.9		6240
42060301	07aug2012	SS	M	0	36.8	11		0
42060302	07aug2012	DS	F	0	36.6	10		0

Gametocyte	*P.malariae (Trophozoites/microliter)*	*P.ovale (Trophozoites/microliter)*	Age/Year
0	0	0	4
0	0	0	4
0	0	0	1
0	0	0	4
0	0	0	4
0	0	0	2
0	0	0	4
0	0	0	3
0	0	0	3
0	0	0	4
0	0	0	2
0	0	0	.8333333
0	0	0	4
0	0	0	2
0	0	0	3
0	0	0	2
0	0	0	4
1	0	0	4
0	0	0	.75
0	0	0	3
0	0	0	2
0	0	0	4
0	0	0	2
0	0	0	2
0	0	0	3
0	0	0	3
0	0	0	4
0	0	0	2
0	0	0	3
0	0	0	4
0	0	0	1
0	0	0	2

0	0	0	4
0	0	0	2
0	0	0	.5833333
0	0	0	2
0	0	0	1
0	0	0	1
0	0	0	1
0	0	0	2
0	0	0	4
1	0	0	2
0	0	0	3
1	0	0	3
0	0	0	1
0	0	0	1
0	0	0	1
0	0	0	2
0	0	0	2
0	0	0	4
0	0	0	2
0	0	0	1
0	0	0	2
0	0	0	2
0	0	0	1
0	0	0	4
12	0	0	.5
0	0	0	3
0	0	0	2
0	0	0	.8333333
0	0	0	3
8	0	0	4
0	0	0	1
0	0	0	1
0	0	0	2
1	0	0	2

4	0	0	4
0	0	0	2
0	0	0	4
0	0	0	4
0	0	0	2
0	0	0	1
0	0	0	.5833333
0	0	0	2
0	0	0	3
0	0	0	2
0	0	0	3
0	0	0	.5
0	0	0	1
0	0	0	4
0	0	0	4
0	0	0	4
0	0	0	4
0	0	0	3
0	0	0	.8333333
0	0	0	4
0	0	0	4
0	0	0	2
0	0	0	.8333333
0	0	0	4
0	0	0	1
9	0	0	3
0	0	0	1
13	0	0	1
0	0	0	4
0	0	0	1
0	0	0	2
0	0	0	3
0	0	0	3
0	0	0	4

0	0	0	2
0	0	0	1
0	0	0	2
0	0	0	.25
0	0	0	3
0	0	0	.8333333
1	0	0	4
0	0	0	2
0	0	0	1
6	0	0	3
0	0	0	3
6	0	0	2
0	0	0	2
0	0	0	3
0	0	0	4
0	0	0	3
0	0	0	3
0	0	0	3
0	0	0	4
0	0	0	4
0	0	0	2
0	0	0	1
0	0	0	3
0	0	0	.4166667
0	0	0	1
0	0	0	.8333333
0	0	0	1
0	0	0	.75
0	0	21760	3
5	0	0	3
0	0	0	2
0	0	0	3
0	0	0	3
0	0	0	4

0	0	0	1
0	0	0	3
0	0	0	2
0	0	0	.9166667
0	0	0	4
			2
1	0	0	4
0	0	0	2
0	0	0	.3333333
0	0	0	2
0	0	0	4
0	0	0	4
0	0	0	3
0	0	0	3
0	0	0	1
0	0	0	2
0	0	0	4
0	0	0	1.5
0	0	0	2
0	0	0	.75
0	0	0	3
7	0	0	4
5	0	0	2
8	0	0	2
0	0	0	2
0	0	0	2
3	0	0	4
0	0	0	4
0	0	0	1.5
0	0	0	4
0	1400	0	2
0	0	0	4
0	0	0	2
0	0	0	4

0	0	0	1
0	0	0	4
0	0	0	4
0	0	0	3
0	0	0	.5
0	0	0	3
0	0	0	4
0	0	0	0
0	0	0	2
0	0	0	4
0	0	0	1
0	0	0	4
0	0	0	2
0	0	0	1
0	0	0	4
0	0	0	4
0	0	0	2
0	0	0	4
0	0	0	1.5
0	0	0	4
0	0	0	.25
0	0	0	3
0	0	0	2
0	0	0	4
0	0	0	4
0	0	0	2
0	0	0	3
0	0	0	.3333333
0	0	0	3
0	0	0	.3333333
0	0	0	2
0	0	0	.75
0	0	0	3
0	0	0	1

0	0	0	3
0	0	0	1
0	0	0	3
0	0	0	1
0	0	0	.5833333
0	0	0	3
0	0	0	.9166667
0	0	0	4
0	0	0	1
0	0	0	.8333333
0	0	0	3
0	0	0	1
0	0	0	4
0	0	0	3
0	0	0	4
0	0	0	2
0	0	0	4
0	0	0	3
0	0	0	1
0	0	0	2
0	0	0	1
0	0	0	2
0	0	0	1
0	0	0	3
0	0	0	1
0	0	0	3
0	0	0	.5
0	0	0	4
0	0	0	2
0	0	0	.3333333
0	0	0	1
0	0	0	4
0	0	0	2
0	0	0	1

0	0	0	4
0	0	0	2
0	0	0	3
0	0	0	2
0	0	0	2
0	0	0	1
0	0	0	4
0	0	0	1
0	0	0	4
0	0	0	1
0	0	0	1
0	0	0	4
0	0	0	1
0	0	0	4
0	0	0	4
0	0	0	1
0	0	0	4
0	0	0	.5
0	0	0	1
0	0	0	3
0	0	0	3
0	0	0	3
0	0	0	4
0	0	0	4
0	0	0	4
0	0	0	1
0	0	0	4
0	0	0	1
0	0	0	3
0	0	0	3
0	0	0	2
0	0	0	3
0	0	0	4
0	0	0	1

0	0	0	.6666667
0	0	0	1
0	0	0	4
0	0	0	4
0	0	0	.25
5	0	0	2
0	0	0	.3333333
2	0	0	4
0	0	0	4
2	0	0	4
0	0	0	2
0	0	0	3
0	0	0	3
0	0	0	1
0	0	0	1
0	0	0	4
0	0	0	1.583333
0	0	240	1
11	0	0	4
0	0	0	.75
0	0	0	1
0	0	0	2
0	0	0	1
0	0	0	2
0	0	0	.5833333
0	0	0	1
0	0	0	3
0	0	0	1
0	0	0	2
0	0	0	3
0	0	0	4
0	0	0	2
0	0	0	3
0	0	0	.5

0	0	0	4
0	0	0	2
0	0	0	1
0	0	0	1
0	0	0	2
0	0	0	3
0	0	0	2
0	0	0	1
0	0	0	3
0	0	0	1
0	0	0	1
1	0	0	4
0	0	0	2
0	0	0	1
0	0	0	4
0	0	0	1
0	0	0	1
2	0	0	4
0	0	0	1
0	0	0	2
1	0	0	4
1	0	0	4
0	0	0	3
0	0	0	1.916667
0	0	0	.75
0	0	0	1
0	0	0	3
0	0	0	1
0	0	0	3
0	0	0	2
0	0	0	4
0	0	0	.25
0	0	0	4
0	0	0	2

0	0	0	1
0	0	0	4
0	0	0	1
0	0	0	2
0	0	0	4
0	0	0	.75
0	0	0	2
0	0	0	1
0	0	0	4
0	0	0	1
0	0	0	4
0	0	0	1
0	0	0	3
23	0	0	2
0	0	0	4
0	0	0	3
0	0	0	1
0	0	0	2
0	0	0	3
0	0	0	1
0	0	0	2
0	0	0	2
0	0	0	3
0	0	0	.8333333
0	0	0	3
0	0	0	.25
0	0	0	3
0	0	0	1
0	0	0	2
0	0	0	4
0	0	0	1
0	0	0	4
0	0	0	3
0	0	0	1

1	0	0	3
0	0	0	2
0	0	0	1
0	0	0	4
0	0	0	2
0	0	0	4
0	0	0	4
0	0	0	2
0	0	0	2
0	0	0	4
0	0	0	4
7	0	0	1
0	0	0	4
0	0	0	2
0	0	0	2
0	0	0	3
0	0	0	3
0	0	0	4
5	0	0	4
3	0	0	4
0	0	0	3
0	0	0	3
0	0	0	2
0	0	0	4
0	0	0	4
0	0	0	1
0	0	0	4
5	0	0	3
0	0	0	3
0	0	0	1
0	0	0	3
0	0	0	.8333333
0	0	0	2
0	0	0	4

0	0	0	2
0	0	0	4
0	0	0	3
0	0	0	1
0	0	0	2
0	0	0	4
1	0	0	1
0	0	0	2
0	0	0	1
0	0	0	3
0	0	0	4
0	0	0	2
0	0	0	.8333333
0	0	0	4
0	0	0	2
0	0	0	4
0	0	0	2
0	480	0	4
0	0	0	2
0	0	0	2
0	0	0	3
0	0	0	4
0	0	12640	2
0	0	0	2
1	0	0	1
0	0	0	2
0	0	0	2
0	0	0	1
0	0	0	2
0	0	0	2
0	0	0	3
0	0	0	2
0	0	0	.3333333
1	0	0	1

0	0	0	2
0	0	0	4
0	0	0	3
0	0	0	2
0	0	0	2
0	0	0	.6666667
0	0	0	3
4	0	0	2
0	0	0	.5
0	0	0	1
0	0	0	4
0	0	0	2
0	0	0	.25
0	0	0	4
0	0	0	4
0	0	0	2
0	0	0	4
0	0	0	1
0	0	0	2
0	0	0	4
0	0	0	1
0	0	0	3
0	0	0	2
0	0	0	1
0	0	0	3
0	0	0	1.166667
0	0	0	4
1	0	0	2
17	0	0	4
0	0	0	2
2	0	0	4
0	0	0	3
4	0	0	3
2	0	0	4

0	0	0	.8333333
0	0	0	2
0	0	0	3
0	0	0	.25
1	0	0	3
0	0	0	.9166667
0	0	0	4
0	0	0	3
0	0	0	1.5
0	0	0	4
0	0	0	4
0	0	0	.4166667
0	0	0	1.5
0	0	0	4
0	0	0	4
0	0	0	.8333333
0	0	0	1
0	0	0	1
0	0	0	3
0	0	0	1
0	0	0	3
0	0	0	2
0	0	0	4
0	0	0	1.25
0	0	0	4
0	0	0	2
0	0	0	2
0	0	0	.3333333
0	0	0	2
0	0	0	1.833333
0	0	0	.5
0	0	0	4
0	0	0	1.416667
0	0	0	3

0	0	0	2
0	0	0	2
0	0	0	4
0	0	0	2
0	0	0	4
0	0	0	3
0	0	0	.25
0	0	0	2
0	0	0	4
0	0	0	.5
0	0	0	3
0	0	0	3
0	0	0	1
0	0	0	3
0	0	0	4
0	0	0	2
0	0	0	1
0	0	0	4
0	0	0	3
0	0	0	2
0	0	0	2
23	0	0	4
0	0	0	1
0	0	0	2
0	0	0	.5833333
0	0	0	2
0	0	0	4
0	0	0	.5
0	0	0	1
0	0	0	3
0	0	0	3
0	0	0	3
0	0	0	.75
11	0	0	2

0	0	0	1.25
0	0	0	3
0	0	0	1
0	0	0	.25
0	0	0	3
0	0	0	.3333333
0	0	0	3
0	0	0	3
0	0	0	.3333333
0	0	0	.5833333
0	0	0	1.5
9	0	0	4
0	0	0	1
0	0	0	4
0	0	0	2
0	0	0	1
0	0	0	3
0	0	0	1.833333
0	0	0	3
0	0	0	1
0	0	0	1.333333
3	0	0	2
0	0	0	4
0	0	0	4
0	0	0	3
0	0	0	4
0	0	0	.8333333
0	0	0	4
0	0	0	.9166667
4	0	0	4
0	0	0	2
0	0	0	2
1	0	0	3
0	0	0	.5

28	0	0	4
7	0	0	2
0	0	0	2
0	6160	0	4
0	0	0	1
0	0	0	.5
0	0	0	2
0	0	0	1.5
2	0	0	4
0	0	0	4
0	0	0	3
0	0	0	2
94	0	0	3
0	0	0	4
0	0	0	1
0	0	0	2
0	0	0	4
0	0	0	.9166667
0	0	0	2
0	0	0	2
0	0	0	1.5
0	0	0	4
0	0	0	2
0	0	0	4
0	0	0	4
33	0	0	2
0	0	0	3
0	0	0	.75
0	0	0	1
0	0	0	4
0	0	0	1
0	0	0	2
0	0	0	4
0	0	0	2

1	0	0	2
2	0	0	4
0	0	0	1
0	0	0	2
0	0	0	2
0	0	0	3
27	0	0	2
0	0	0	.3333333
0	0	0	1
0	0	0	1
0	0	0	2
0	0	0	4
0	0	0	2
0	0	0	2
36	0	0	2
0	0	0	3
0	0	0	2
0	0	0	2
53	0	0	2
0	0	0	3
0	0	0	1
0	0	0	3
0	0	0	4
0	0	0	2
1	0	0	3
0	0	0	3
0	0	0	2
0	0	0	1
0	0	0	3
0	0	0	1
			.3333333
0	0	0	2
0	0	0	.25
0	0	0	4

0	0	0	.25
0	0	0	3
0	0	0	1
0	0	0	4
0	0	0	4
0	0	0	.9166667
0	0	0	3
0	0	0	3
0	0	0	4
0	0	0	2
0	0	0	4
1	0	0	3
0	0	0	.8333333
0	0	0	.5
0	0	0	4
0	0	0	2
0	0	0	4
0	0	0	.3333333
