# Supplementary material for: Seasonal Malaria Chemoprevention with Sulphadoxine-Pyrimethamine and Amodiaquine Selects Pfdhfr-dhps Quintuple Mutant Genotype in Mali
Source: PLoS One. 2016 Sep 23;11(9):e0162718. doi: 10.1371/journal.pone.0162718 (PMC5035027; doi:10.1371/journal.pone.0162718)
Supplement: S2 File — (PDF) [file pone.0162718.s002.pdf]

ID	Dates	*PfDHPS540*	*PfDHPS437*	*PfDHFR51*	*PfDHFR59*	*PfDHFR108*	*PFCRT-76*	*Pfmdr1-86*
22 06 05 01	04--08--12	1	2	1	2	2	2	2
22 06 04 01	04--08--12	1	1	2	2	2	2	2
22 06 03 01	04--08--12	1	2	2	2	2	2	1
22 06 02 01	04--08--12	1	2	2	2	0	2	1
33 13 01 01	05--08--12	2	2	2	2	2	2	2
33 12 01 01	05--08--12	2	2	1	1	1	2	1
33 11 01 01	05--08--12	1	2	2	2	2	2	1
33 10 03 01	05--08--12	1	2	2	2	2	2	1
33 10 02 01	05--08--12	1	1	2	2	2	1	1
33 10 01 01	05--08--12	1	2	2	2	2	2	2
33 08 02 01	05--08--12	1	1	2	2	2	2	2
33 06 01 01	05--08--12	1	1	2	2	2	1	1
33 05 01 01	05--08--12	2	2	1	1	1	1	1
33 03 03 01	05--08--12	1	2	2	2	2	1	1
33 03 02 02	05--08--12	1	2	0	0	0	2	1
33 03 01 01	05--08--12	1	2	2	2	2	1	1
33 02 01 01	05--08--12	1	2	2	2	2	1	2
33 01 04 02	05--08--12	1	2	2	2	2	0	2
33 01 04 01	05--08--12	1	2	0	0	0	0	1
33 01 02 01	05--08--12	1	2	2	2	2	2	2
29 08 02 01	05--08--12	1	2	0	0	2	0	1
29 08 01 01	05--08--12	1	1	0	0	2	2	1
29 06 01 02	05--08--12	1	2	0	0	2	2	1
29 06 01 01	05--08--12	1	2	0	0	2	2	1
29 05 05 02	05--08--12	1	2	0	0	2	2	1
29 05 05 01	05--08--12	1	1	0	0	2	2	1
29 05 04 02	05--08--12	1	1	2	2	2	2	1
29 05 04 01	05--08--12	1	1	0	0	2	2	1
29 05 03 01	05--08--12	1	2	2	1	0	2	1
29 05 02 02	05--08--12	1	2	2	2	2	2	1
29 05 02 01	05--08--12	1	2	2	2	2	2	1

29 05 01 04	05--08--12	1	2	2	2	2	2	1
29 05 01 01	05--08--12	1	2	2	2	2	2	1
29 04 01 01	05--08--12	1	2	1	2	0	2	1
21 06 07 01	05--08--12	1	2	2	2	2	2	1
21 06 04 01	05--08--12	1	2	2	2	0	2	1
21 06 01 01	05--08--12	1	2	2	2	2	1	1
21 05 01 01	05--08--12	1	2	2	2	1	2	1
21 03 03 01	05--08--12	1	2	2	2	2	2	1
21 01 02 01	05--08--12	1	1	2	2	0	2	1
11 06 01 01	05--08--12	1	2	1	1	1	2	2
11 04 03 01	05--08--12	1	1	1	1	0	2	1
11 02 01 01	05--08--12	1	1	1	1	1	1	1
11 01 11 01	05--08--12	1	1	2	2	2	2	1
11 01 08 01	05--08--12	1	1	2	2	2	2	1
11 01 07 01	05--08--12	1	2	2	2	2	2	1
11 01 06 01	05--08--12	1	0	2	2	2	2	2
108 05 01	05--08--12	1	0	1	1	1	2	1
108 01 02	05--08--12	1	2	2	2	2	2	1
107 02 01 Bis	05--08--12	1	2	2	2	0	2	2
107 02 01	05--08--12	1	2	1	1	0	2	2
102 02 01	05--08--12	1	2	1	1	1	2	2
102 01 01	05--08--12	1	2	0	0	1	2	2
101 03 01	05--08--12	1	2	1	2	2	2	1
27 09 01 01	06--08--12	1	2	2	2	0	2	1
27 08 02 01	06--08--12	1	1	2	2	0	2	1
27 08 01 01	06--08--12	1	1	2	2	0	2	1
27 06 02 01	06--08--12	1	2	2	2	0	2	1
27 06 01 01	06--08--12	1	2	2	2	2	1	2
27 05 02 01	06--08--12	1	2	2	2	2	1	2
27 01 01 01	06--08--12	1	2	2	2	0	2	1
26 06 03 01	06--08--12	1	2	2	2	2	2	1
26 06 02 01	06--08--12	1	1	2	2	0	2	1

26 06 01 01	06--08--12	1	2	2	2	2	1	1
26 05 05 01	06--08--12	1	2	2	2	0	2	1
26 05 04 01	06--08--12	1	2	2	2	2	2	1
26 05 03 01	06--08--12	1	1	2	2	0	1	1
26 05 02 01	06--08--12	1	2	2	2	0	2	1
26 05 01 01	06--08--12	1	2	2	2	0	1	1
26 04 05 01	06--08--12	1	2	2	2	0	2	1
26 04 03 01	06--08--12	1	1	0	0	0	0	1
26 04 02 02	06--08--12	1	2	0	0	0	0	1
26 04 02 01	06--08--12	1	2	0	0	0	0	1
26 04 01 02	06--08--12	1	1	0	0	0	0	1
26 04 01 01	06--08--12	1	2	0	0	0	2	1
26 03 05 01	06--08--12	1	2	0	0	0	0	2
26 03 04 01	06--08--12	1	2	0	0	0	0	1
26 03 03 01	06--08--12	1	2	2	2	0	0	1
26 03 02 01	06--08--12	1	1	1	2	0	0	1
26 02 03 01	06--08--12	1	2	2	2	0	0	1
26 01 03 01	06--08--12	1	1	2	2	0	0	2
26 01 02 01	06--08--12	1	1	2	2	0	2	1
26 01 01 01	06--08--12	1	2	2	2	0	0	1
23 07 02 01	06--08--12	1	2	2	2	0	0	1
23 05 02 01	06--08--12	1	2	2	2	0	0	1
23 04 01 01	06--08--12	1	2	2	2	2	2	2
23 01 02 01	06--08--12	1	1	2	2	2	2	1
12 05 05 01	06--08--12	1	1	1	2	2	1	2
12 05 03 01	06--08--12	1	1	1	1	0	2	2
12 05 02 02	06--08--12	1	1	1	1	1	2	1
12 04 01 01	06--08--12	1	1	2	2	2	2	1
12 03 02 02	06--08--12	1	1	2	2	2	2	1
12 02 04 02	06--08--12	1	1	2	2	0	2	2
12 02 04 01	06--08--12	1	1	2	2	2	2	2
12 02 03 01	06--08--12	1	1	1	1	1	1	2

12 02 02 01	06--08--12	1	2	2	2	0	2	2
12 02 01 01	06--08--12	1	1	2	2	2	1	2
112 02 01	06--08--12	1	2	2	2	2	1	1
110 02 01	06--08--12	1	1	2	2	2	2	1
110 01 01	06--08--12	1	1	1	1	1	2	1
06 06 02 01	06--08--12	1	1	2	2	2	1	1
06 05 01 01	06--08--12	1	1	2	2	2	2	1
06 04 03 01	06--08--12	1	1	2	2	2	2	2
06 04 01 01	06--08--12	1	2	2	2	2	1	1
06 03 03 01	06--08--12	1	1	2	2	2	1	1
06 03 01 01	06--08--12	1	2	2	2	2	2	1
06 02 02 02	06--08--12	1	1	2	2	2	2	2
06 01 02 01	06--08--12	1	2	1	2	2	2	1
06 01 01 02	06--08--12	1	2	1	1	1	2	2
42 06 02 02	07--08--12	1	2	1	1	1	1	1
42 06 02 01	07--08--12	1	2	2	2	2	1	1
42 05 03 01	07--08--12	1	2	2	2	2	1	1
42 04 01 01	07--08--12	1	1	2	2	2	1	1
42 03 04 01	07--08--12	1	1	2	2	2	1	2
42 03 01 01	07--08--12	2	1	2	2	2	2	2
42 02 05 01	07--08--12	1	2	2	2	2	2	2
42 02 04 02	07--08--12	1	1	2	2	2	2	1
42 02 04 01	07--08--12	1	1	2	2	2	2	1
42 02 02 02	07--08--12	1	2	2	2	2	2	1
40 10 02 01	07--08--12	1	2	2	2	2	2	2
40 10 01 01	07--08--12	1	2	2	2	2	1	1
40 09 03 01	07--08--12	1	2	2	2	2	1	2
40 09 01 01	07--08--12	1	1	2	2	2	2	1
40 07 01 02	07--08--12	1	1	2	2	2	2	1
40 07 01 01	07--08--12	1	1	2	2	2	2	2
40 06 01 01	07--08--12	1	2	2	2	2	2	1
40 05 01 01	07--08--12	1	2	2	2	2	1	1

40 02 01 01	07--08--12	1	2	2	2	2	1	2
40 01 08 03	07--08--12	1	2	2	2	2	2	1
40 01 08 02	07--08--12	1	1	2	2	2	2	2
40 01 07 01	07--08--12	1	1	2	2	2	2	1
40 01 06 01	07--08--12	1	2	2	2	2	2	2
40 01 03 03	07--08--12	1	2	2	2	2	2	1
40 01 03 02	07--08--12	1	2	1	1	1	1	1
40 01 03 01	07--08--12	1	1	2	2	2	2	1
40 01 02 02	07--08--12	1	2	2	2	2	1	1
40 01 02 01	07--08--12	1	1	2	2	2	1	1
36 15 01 01	07--08--12	2	1	2	2	2	2	1
36 08 01 02	07--08--12	1	2	2	2	2	2	1
36 06 01 01	07--08--12	1	2	2	2	2	2	1
36 04 02 01	07--08--12	1	2	2	2	2	2	1
36 02 01 01	07--08--12	1	2	2	2	2	2	1
36 01 02 01	07--08--12	1	1	2	2	2	2	1
36 01 01 01	07--08--12	1	2	2	2	2	1	1
38 12 01 02	08--08--12	1	2	2	2	2	2	1
38 12 01 01	08--08--12	1	1	2	2	2	2	1
38 11 02 01	08--08--12	1	2	2	2	2	1	2
38 11 01 01	08--08--12	1	1	2	2	2	1	1
38 10 01 01	08--08--12	1	1	2	2	2	2	1
38 09 02 01	08--08--12	1	1	2	2	2	1	2
38 09 01 03	08--08--12	1	2	2	2	2	1	1
38 09 01 02	08--08--12	1	1	2	2	2	1	1
38 09 01 01	08--08--12	1	2	2	2	2	1	1
38 08 02 02	08--08--12	1	1	2	2	2	1	1
38 08 01 01	08--08--12	1	2	2	2	2	1	1
38 07 01 02	08--08--12	1	1	1	1	1	1	2
38 06 02 02	08--08--12	1	2	2	2	2	1	1
38 06 02 01	08--08--12	1	2	2	2	2	2	1
38 06 01 01	08--08--12	1	2	2	2	2	1	1

38 05 01 01	08--08--12	1	1	2	2	2	1	1
38 04 03 01	08--08--12	1	2	2	2	2	1	1
38 04 01 02	08--08--12	1	1	2	2	2	2	1
38 03 01 01	08--08--12	1	1	2	2	2	2	1
38 01 01 02	08--08--12	1	1	2	2	2	2	1
38 01 01 01	08--08--12	1	2	2	2	2	2	1
13 08 05 01	08--08--12	1	1	2	2	0	2	2
08 08 02 01	08--08--12	1	2	2	2	2	2	1
08 08 01 02	08--08--12	0	1	2	2	2	2	2
08 08 01 01	08--08--12	1	0	2	2	2	2	1
08 07 01 01	08--08--12	1	2	2	2	2	2	1
08 06 02 02	08--08--12	2	1	2	2	2	1	1
08 06 02 01	08--08--12	1	2	2	2	0	2	1
08 05 01 01	08--08--12	1	1	2	2	2	2	1
08 04 11 01	08--08--12	1	0	2	2	0	1	2
08 04 10 01	08--08--12	1	1	2	2	2	1	1
08 04 09 01	08--08--12	1	1	2	2	2	1	1
08 04 08 01	08--08--12	1	2	2	2	2	1	1
08 04 07 01	08--08--12	1	2	2	2	2	2	1
08 04 06 04	08--08--12	1	2	2	2	2	1	1
08 04 06 02	08--08--12	1	2	2	2	2	1	1
08 04 03 02	08--08--12	1	2	2	2	2	1	1
08 04 03 01	08--08--12	1	2	2	2	2	2	1
08 04 02 01	08--08--12	1	1	1	2	2	1	1
08 03 01 01	08--08--12	1	2	2	2	2	1	2
08 02 01 01	08--08--12	1	2	2	2	2	1	2
08 01 02 01	08--08--12	1	0	2	2	2	2	2
08 01 01 02	08--08--12	1	1	2	2	2	2	2
08 01 01 01	08--08--12	1	2	2	2	0	2	2
34 08 02 01	09--08--12	1	1	2	2	0	2	2
19 07 01 01	09--08--12	1	2	2	2	2	2	1
17 07 02 02	09--08--12	1	2	2	2	2	2	2
