# Supplementary material for: Seasonal Malaria Chemoprevention with Sulphadoxine-Pyrimethamine and Amodiaquine Selects Pfdhfr-dhps Quintuple Mutant Genotype in Mali
Source: PLoS One. 2016 Sep 23;11(9):e0162718. doi: 10.1371/journal.pone.0162718 (PMC5035027; doi:10.1371/journal.pone.0162718)
Supplement: S3 File — (PDF) [file pone.0162718.s003.pdf]

ID	Date	Sex	Age (years)	Age (months)	Age/Month	Fever History	Température	Hemoglobin
1001	24-juin-14	F		59	59	0	36.2	11.3
1002	24-juin-14	M	1		12	0	36.8	11.3
1003	24-juin-14	F	1	7	19	0	36.1	10.2
1004	24-juin-14	F	3		36	0	37.5	12.2
1005	24-juin-14	M		17	17	1	36.9	11.5
1006	24-juin-14	M	4		48	0	36.1	10.8
1007	24-juin-14	M	4		48	0	36.8	10.4
1008	24-juin-14	F	2	3	27	0	36.1	11.0
1009	24-juin-14	M	3		36	0	36.2	12.8
1010	24-juin-14	M	2	4	28	1	37.5	10.4
1011	24-juin-14	F		59	59	1	36.2	12.6
1012	24-juin-14	M	4		48	0	37.0	12.2
1013	24-juin-14	M	1		12	0	37.0	10.1
1014	24-juin-14	F	3		36	0	36.1	9.8
1015	24-juin-14	M	3		36	0	36.8	12.0
1016	24-juin-14	M	2		24	0	36.7	11.8
1017	24-juin-14	M		28	28	0	36.2	10.8
1018	24-juin-14	F	3		36	1	36.7	10.2
10181	24-juin-14	F	3		36	0	36.5	12.5
1019	24-juin-14	M		5	5	0	36.2	11.0
1020	24-juin-14	M		5	5	0	36.7	9.4
1021	24-juin-14	M	1		12	1	37.1	9.9
1022	24-juin-14	F	4		48	0	36.2	12.5
1023	24-juin-14	M	1		12	1	36.4	9.4
1024	24-juin-14	F	4		48	0	36.5	11.3
1025	24-juin-14	F	1		12	1	36.2	10.5
1026	24-juin-14	M	1		12	0	36.2	11.5
1027	24-juin-14	F	1		12	0	36.4	9.9
1028	24-juin-14	F		59	59	0	36.4	11.6
1029	24-juin-14	M		18	18	0	36.3	10.4
1030	24-juin-14	M		18	18	0	36.2	7.6

1031	24-juin-14	M	2		24	0	36.2	11.7
1032	24-juin-14	M	4		48	0	37.0	10.2
1033	24-juin-14	F	3		36	0	36.8	10.5
1034	24-juin-14	M		11	11	0	36.7	13.3
1035	24-juin-14	F		59	59	0	37.1	12.5
1036	24-juin-14	M		17	17	1	36.4	10.4
1037	24-juin-14	M	3		36	0	36.3	11.0
1038	24-juin-14	M		59	59	0	36.7	12.0
1039	24-juin-14	F		10	10	0	36.1	11.9
1040	24-juin-14	F		10	10	0	36.1	10.7
1041	24-juin-14	M	2		24	0	36.2	8.6
1044	24-juin-14	M	1		12	0	36.3	10.5
1045	24-juin-14	M	3		36	0	36.1	11.8
1046	24-juin-14	M		6	6	0	36.3	10.8
1047	24-juin-14	M		9	9	0	36.9	9.5
1048	24-juin-14	M	1		12	0	36.6	10.9
1049	24-juin-14	F	4		48	0	36.2	12.5
1050	24-juin-14	F	3		36	1	37.0	10.4
1052	24-juin-14	M	3		36	0	37.1	11.8
1053	24-juin-14	F		59	59	0	36.9	10.8
1054	24-juin-14	F	2		24	0	36.8	7.7
1057	24-juin-14	M	3		36	0	36.7	11.9
1058	24-juin-14	M		17	17	0	36.7	8.1
1059	24-juin-14	M		4	4	0	36.7	10.1
1060	24-juin-14	M		10	10	1	36.5	9.6
1061	24-juin-14	M		7	7	0	36.4	11.0
1062	24-juin-14	F	1		12	0	37.1	10.3
1064	24-juin-14	M	3		36	0	35.7	9.6
1065	24-juin-14	F	3		36	0	37.0	10.9
1066	24-juin-14	F	1		12	0	36.2	9.9
1067	24-juin-14	F	4		48	0	36.9	10.9
1068	24-juin-14	M		59	59	0	36.2	11.8

1069	24-juin-14	M		59	59	0	36.7	12.5
1070	24-juin-14	F	3		36	0	36.4	10.8
1071	24-juin-14	M		59	59	0	36.4	11.3
1072	24-juin-14	F	3		36	0	36.3	7.8
1073	24-juin-14	M	4		48	0	37.3	12.3
1074	24-juin-14	F	2		24	0	36.7	12.6
1075	24-juin-14	F	3		36	0	37.0	10.1
1076	24-juin-14	F	1		12	1	36.4	10.2
1077	24-juin-14	M	2		24	0	36.2	10.7
1078	24-juin-14	M		3	3	0	36.3	9.0
1079	24-juin-14	M	4		48	0	37.2	9.9
1080	24-juin-14	F		59	59	0	36.7	10.6
1081	24-juin-14	M	3		36	0	36.4	10.3
1101	25-juin-14	F	4		48	0	36.1	10.5
1102	25-juin-14	F	3		36	0	36.1	11.6
1103	25-juin-14	F	2		24	0	35.9	11.9
1104	25-juin-14	F	1		12	0	36.2	10.6
1105	25-juin-14	M		5	5	0	36.7	9.0
1106	25-juin-14	M	3		36	0	36.5	11.5
1107	25-juin-14	M	3		36	0	36.8	10.4
1108	25-juin-14	F	2		24	0	36.3	9.9
1109	25-juin-14	F	3		36	0	37.0	10.6
1110	25-juin-14	M		59	59	0	36.2	10.9
1111	25-juin-14	F		8	8	0	36.7	11.3
1112	25-juin-14	M	4		48	0	36.1	11.1
1113	25-juin-14	M	3		36	0	36.0	12.4
1114	25-juin-14	M	3		36	0	35.8	8.3
1115	25-juin-14	M		59	59	0	36.8	10.2
1116	25-juin-14	F	2		24	0	36.1	11.2
1117	25-juin-14	F	2		24	0	36.4	11.2
1118	25-juin-14	M	4		48	0	36.0	13.0
1119	25-juin-14	F		59	59	0	36.5	9.7

1120	25-juin-14	F		59	59	0	36.0	13.9
1121	25-juin-14	F	4		48	0	36.1	12.7
1122	25-juin-14	M	1		12	0	36.4	9.5
1123	23-juin-14	F	3		36	0	36.1	11.0
1124	25-juin-14	M	2		24	0	36.2	10.4
1125	25-juin-14	M	2		24	0	36.3	10.4
1126	25-juin-14	F		18	18	0	36.1	12.1
1127	25-juin-14	F		7	7	0	36.5	10.9
1128	25-juin-14	M	4		48	0	36.2	11.5
1129	25-juin-14	F	2		24	0	36.6	8.0
1130	25-juin-14	M		30	30	0	36.5	10.4
1131	25-juin-14	M	4		48	0	36.1	11.3
1132	25-juin-14	F		59	59	0	37.0	12.6
1133	25-juin-14	F		59	59	0	37.0	9.8
1134	25-juin-14	M		59	59	0	36.3	12.2
1135	25-juin-14	F		17	17	0	36.1	11.7
1136	25-juin-14	M	1		12	0	36.3	8.5
1137	25-juin-14	M		59	59	0	36.2	11.4
1138	25-juin-14	M		59	59	0	36.6	12.3
1139	25-juin-14	M	4		48	0	36.8	11.5
1140	25-juin-14	M	3		36	0	36.1	12.5
1141	25-juin-14	F	2		24	0	36.8	11.0
1142	25-juin-14	F	1		12	0	36.2	10.5
1143	25-juin-14	F	4		48	0	36.4	10.5
1144	25-juin-14	M	3		36	0	36.6	11.3
1145	25-juin-14	M	1		12	0	36.9	9.5
1146	25-juin-14	M	3		36	0	36.1	11.4
1147	25-juin-14	M	4		48	0	36.6	10.4
1148	25-juin-14	F		59	59	0	36.2	12.5
1149	25-juin-14	F		16	16	0	36.6	9.9
1150	25-juin-14	F		20	20	0	36.5	9.0
1151	25-juin-14	M	2		24	0	36.9	10.6

1152	25-juin-14	F		59	59	0	36.2	12.1
1153	25-juin-14	F	2		24	0	36.6	12.5
1154	25-juin-14	F	4		48	0	36.7	11.6
1155	25-juin-14	M	4		48	0	36.4	10.4
1156	25-juin-14	F		59	59	0	36.1	11.3
1157	25-juin-14	M	3		36	0	36.7	10.0
1158	25-juin-14	M	4		48	0	36.7	11.4
1159	25-juin-14	F		27	27	0	36.1	10.1
1160	25-juin-14	F	4		48	0	36.2	10.9
1161	25-juin-14	M	2		24	0	36.9	10.0
1162	25-juin-14	F	3		36	1	36.7	11.4
1163	25-juin-14	M	3		36	0	37.4	12.1
1164	25-juin-14	F	4		48	0	37.0	11.7
1165	25-juin-14	F		59	59	0	36.1	12.1
2001	22-juin-14	F	2		24	0	35.6	10.2
2002	22-juin-14	F		59	59	0	35.9	13.0
2003	22-juin-14	F	3		36	0	35.8	9.6
2004	22-juin-14	M		59	59	0	35.9	10.5
2005	22-juin-14	F	3		36	0	36.2	10.6
2006	22-juin-14	F	2		24	0	35.6	11.2
2007	22-juin-14	F	2	5	29	0	36.4	10.9
2008	22-juin-14	F	2		24	0	36.7	12.0
2009	22-juin-14	F	3		36	0	37.0	10.9
2010	22-juin-14	F		59	59	0	36.7	11.0
2011	22-juin-14	M	4		48	0	36.6	9.7
2012	22-juin-14	F	4		48	0	36.1	9.6
2013	22-juin-14	M	4		48	0	36.7	11.9
2014	22-juin-14	M	2		24	0	36.4	9.5
2015	22-juin-14	M	4		48	0	36.1	10.0
2016	22-juin-14	F	3		36	0	35.7	11.0
2017	22-juin-14	F	3	4	40	0	36.2	10.7
2018	22-juin-14	F	1		12	0	36.4	11.8

2019	22-juin-14	M	1		12	1	36.4	8.9
2020	22-juin-14	M		59	59	0	36.6	11.6
2021	22-juin-14	F	4		48	0	36.4	10.6
2022	22-juin-14	F	3		36	0	36.1	11.3
2023	22-juin-14	M		59	59	0	35.9	11.2
2024	22-juin-14	M		54	54	0	36.7	12.3
2025	22-juin-14	F	3		36	1	35.6	12.3
2026	22-juin-14	F	3		36	0	35.9	11.1
2027	22-juin-14	M	3		36	0	36.4	9.9
2028	22-juin-14	M	3	6	42	0	36.2	11.7
2029	22-juin-14	M		59	59	0	36.4	10.3
2030	22-juin-14	M	3		36	1	35.6	13.2
2031	22-juin-14	M	4		48	0	36.8	11.2
2032	22-juin-14	F	3		36	0	36.1	10.7
2033	22-juin-14	M		10	10	0	37.1	10.0
2034	22-juin-14	F		59	59	0	36.1	10.6
2035	22-juin-14	M	1		12	0	35.9	10.4
2036	22-juin-14	F		59	59	0	35.9	10.9
2037	22-juin-14	F		8	8	0	36.6	11.0
2038	22-juin-14	M	4		48	0	36.1	9.9
2039	22-juin-14	F	3		36	0	35.8	11.5
2040	22-juin-14	M	1		12	0	35.9	11.3
2041	22-juin-14	F	3		36	0	35.6	12.8
2042	22-juin-14	F	2		24	0	36.4	11.0
2043	22-juin-14	F		59	59	0	35.6	9.2
2044	22-juin-14	F		5	5	0	36.7	10.9
2045	22-juin-14	F	4		48	0	36.3	10.1
2046	22-juin-14	M	3		36	0	36.2	10.9
2047	22-juin-14	M	3		36	0	35.9	11.3
2048	22-juin-14	M	2		24	0	35.6	10.8
2049	22-juin-14	M	4		48	0	36.4	10.1
2050	22-juin-14	M	4		48	0	36.4	10.2

2051	22-juin-14	F		4	4	0	36.1	10.9
2052	22-juin-14	M	1		12	1	36.5	11.7
2053	22-juin-14	F	3		36	0	36.5	11.6
2054	22-juin-14	M	3		36	0	35.5	11.7
2055	22-juin-14	F		7	7	1	36.5	11.3
2056	22-juin-14	F		59	59	0	36.4	10.9
2057	22-juin-14	M	3		36	0	37.1	11.4
2058	22-juin-14	F	4		48	0	36.4	12.0
2059	22-juin-14	M		7	7	0	36.4	10.2
2060	22-juin-14	F		7	7	1	36.9	10.8
2061	22-juin-14	F	1	6	18	0	36.4	11.0
2062	22-juin-14	M		4	4	0	37.3	12.0
2063	22-juin-14	F	3		36	0	35.7	12.4
2064	22-juin-14	F		59	59	0	36.4	11.1
2065	22-juin-14	M		10	10	0	36.9	11.1
2066	22-juin-14	M	3		36	0	36.0	10.6
2067	22-juin-14	F		59	59	1	35.6	10.5
2068	22-juin-14	F	2		24	0	36.6	11.1
3001	21-juin-14	F	3		36	0	36.8	8.0
3002	21-juin-14	F		59	59	1	37.1	12.9
3003	21-juin-14	F	2		24	0	37.3	11.2
3004	21-juin-14	M		8	8	1	36.4	11.1
3005	21-juin-14	M		7	7	1	36.8	9.8
3006	21-juin-14	M	3		36	1	36.2	9.4
3007	21-juin-14	M		59	59	1	35.7	12.0
3008	21-juin-14	F		59	59	1	36.1	9.7
3009	21-juin-14	F	1	2	14	0	37.2	11.0
3010	21-juin-14	F		14	14	1	36.7	10.9
3011	21-juin-14	M	4		48	0	36.1	9.3
3012	21-juin-14	M	2		24	0	37.2	9.1
3013	21-juin-14	F		5	5	0	36.7	10.7
3014	21-juin-14	M	3		36	0	36.2	12.0

3015	21-juin-14	F	2		24	1	37.1	10.2
3016	21-juin-14	F		59	59	1	36.3	11.2
3017	21-juin-14	F	3		36	1	37.0	10.3
3018	21-juin-14	F	4		48	1	36.6	12.6
3019	21-juin-14	F	2		24	1	37.3	11.7
3020	21-juin-14	F	4		48	0	36.2	11.2
3021	21-juin-14	M	2		24	0	37.1	8.5
3022	21-juin-14	F		30	30	0	36.7	11.3
3023	21-juin-14	F	2		24	1	37.5	11.6
3024	21-juin-14	M	4		48	0	36.7	12.8
3025	21-juin-14	M	2	6	30	0	36.7	10.8
3026	21-juin-14	F		4	4	0	37.2	10.6
3027	21-juin-14	M		4	4	0	36.6	9.1
3028	21-juin-14	M	3		36	0	36.5	11.7
3029	21-juin-14	M	3		36	1	36.7	9.9
3030	21-juin-14	M		3	3	1	37.1	11.1
3031	21-juin-14	F	2		24	0	37.4	11.0
3032	21-juin-14	F	2		24	1	37.5	11.0
3033	21-juin-14	F		5	5	0	36.1	11.2
3034	21-juin-14	F	3		36	0	36.9	10.8
3035	21-juin-14	M	3		36	0	36.6	11.9
3036	21-juin-14	M	4		48	0	37.2	11.5
3037	21-juin-14	F	3		36	0	36.8	11.8
3038	21-juin-14	M		3	3	0	36.9	11.2
3039	21-juin-14	F	4		48	0	37.1	13.0
3040	21-juin-14	F	4		48	0	37.3	9.1
3041	21-juin-14	M		5	5	0	37.2	9.6
3043	21-juin-14	F	1		12	1	37.4	8.3
3044	21-juin-14	M	3		36	0	36.9	10.5
3045	21-juin-14	M		59	59	0	37.3	10.1
3046	21-juin-14	M	2		24	0	36.7	11.0
3047	21-juin-14	M		59	59	0	36.8	10.4

3048	21-juin-14	F	1		12	0	36.8	10.0
3049	21-juin-14	M	4		48	0	36.7	11.4
3050	21-juin-14	F	2		24	0	37.1	12.0
3051	21-juin-14	F	1		12	0	36.1	11.8
3052	21-juin-14	M		3	3	0	36.9	10.8
3053	21-juin-14	M	3		36	0	36.7	12.6
3054	21-juin-14	M	3		36	1	37.2	12.1
3055	21-juin-14	M		59	59	1	37.6	11.5
3056	21-juin-14	M		59	59	1	36.9	11.9
3057	21-juin-14	M	4		48	1	37.2	10.3
3058	21-juin-14	M		59	59	0	37.2	10.4
3059	21-juin-14	F	2		24	0	36.2	12.5
3060	21-juin-14	M	2		24	1	35.7	11.4
3061	21-juin-14	M		17	17	0	36.9	10.5
3062	21-juin-14	F	4		48	0	37.2	11.2
3063	21-juin-14	F	3		36	0	36.6	10.0
3064	21-juin-14	M	2		24	0	37.4	11.3
3065	21-juin-14	M	4		48	0	36.6	11.4
3066	21-juin-14	F	1		12	0	36.1	11.7
3067	21-juin-14	F	1		12	0	36.8	11.3
3068	21-juin-14	M	3		36	0	36.8	11.4
4001	28-juin-14	F	3		36	0	36.7	11.7
4002	28-juin-14	F	2		24	0	35.6	10.0
4007	28-juin-14	M		9	9	1	36.4	9.3
4008	28-juin-14	F	4		48	1	35.9	9.8
4009	28-juin-14	M	4	5	53	0	36.2	11.0
4010	28-juin-14	M		59	59	0	36.4	10.5
4011	28-juin-14	F	4		48	0	35.8	8.0
4012	28-juin-14	F	3	6	42	0	36.3	10.3
4013	28-juin-14	M	2		24	0	36.4	8.2
4014	28-juin-14	M		7	7	0	37.3	7.5
4015	28-juin-14	M	3	4	40	0	36.7	12.7

4016	28-juin-14	M		3	3	0	36.9	9.9
4017	28-juin-14	M	4		48	0	36.8	9.2
4018	28-juin-14	F		24	24	0	36.7	12.1
4019	28-juin-14	M	3		36	0	36.7	11.8
4020	28-juin-14	F	2		24	0	35.9	9.8
4021	28-juin-14	M	2		24	0	36.6	11.5
4022	28-juin-14	M	1		12	0	36.9	10.6
4023	28-juin-14	M	3		36	0	36.5	11.3
4024	28-juin-14	F	4		48	0	36.9	12.0
4025	28-juin-14	F	4		48	1	36.7	12.9
4026	28-juin-14	M	2		24	0	35.9	11.9
4027	28-juin-14	M		6	6	0	37.0	10.6
4029	28-juin-14	M		9	9	1	35.9	10.4
4030	28-juin-14	F	3		36	0	36.2	12.2
4031	28-juin-14	F	4		48	1	36.3	11.7
4032	28-juin-14	F		7	7	0	36.4	10.1
4033	28-juin-14	F	2		24	1	36.8	9.8
4034	28-juin-14	F	3		36	0	36.4	11.9
4035	28-juin-14	F	3		36	0	36.1	12.5
4038	28-juin-14	M		10	10	1	36.8	10.5
4039	28-juin-14	M	4		48	1	36.3	12.1
4040	28-juin-14	M	4		48	0	36.1	11.2
4041	28-juin-14	F		15	15	0	36.8	10.9
4042	28-juin-14	M	4		48	1	37.0	11.0
4043	28-juin-14	M	3	6	42	0	37.2	10.7
4044	28-juin-14	M	1		12	0	36.5	11.1
4045	28-juin-14	F		59	59	0	37.3	8.7
4046	28-juin-14	M		14	14	0	36.1	8.5
4047	28-juin-14	F	1		12	0	36.4	10.7
4048	28-juin-14	F	4		48	0	36.2	10.7
4049	28-juin-14	F	2		24	0	36.9	9.5
4050	28-juin-14	M		8	8	0	36.7	10.7

4051	28-juin-14	M		59	59	0	36.6	12.2
4052	28-juin-14	F		14	14	0	36.1	11.2
4053	28-juin-14	M	4		48	0	36.7	10.4
4054	28-juin-14	M	3		36	0	36.7	10.9
4055	28-juin-14	F	3	6	42	0	36.1	12.5
4056	28-juin-14	M	2		24	0	36.3	10.1
4057	28-juin-14	M		10	10	0	37.3	8.8
4058	28-juin-14	M		59	59	0	37.1	11.2
4059	28-juin-14	F	4		48	0	36.9	10.5
4060	28-juin-14	M		59	59	0	36.7	11.5
4061	28-juin-14	F	1		12	0	36.3	11.1
4062	28-juin-14	F	1		12	1	36.2	10.4
4063	28-juin-14	F	2		24	0	36.6	11.6
4064	28-juin-14	F	2		24	0	36.5	12.2
4065	28-juin-14	F	2		24	0	36.8	11.7
4066	28-juin-14	M		59	59	0	36.9	11.7
4067	28-juin-14	F	1	3	15	0	35.7	10.8
4068	28-juin-14	F	2		24	1	36.8	10.6
5001	29-juin-14	F		3	3	0	36.0	12.7
5002	29-juin-14	F		18	18	0	36.2	11.3
5003	29-juin-14	F		59	59	0	36.9	12.1
5004	29-juin-14	F	3		36	0	36.1	9.9
5005	29-juin-14	F	4		48	0	35.8	10.3
5006	29-juin-14	F	3		36	0	36.9	9.4
5007	29-juin-14	F	3		36	0	36.8	10.6
5008	29-juin-14	F		8	8	1	35.9	9.2
5009	29-juin-14	F	3		36	1	36.9	10.5
5010	29-juin-14	M		59	59	0	36.1	11.3
5011	29-juin-14	F		59	59	0	36.4	10.2
5012	29-juin-14	M	3		36	0	36.7	10.9
5013	29-juin-14	F		30	30	0	36.8	11.1
5014	29-juin-14	M	2		24	0	36.6	10.5

5015	29-juin-14	F		3	3	0	36.5	12.6
5017	29-juin-14	M		59	59	0	36.5	11.0
5018	29-juin-14	F		8	8	0	36.1	10.7
5019	29-juin-14	F		59	59	0	36.2	10.1
5020	29-juin-14	F	4		48	0	36.6	11.7
5021	29-juin-14	F	2		24	0	36.6	10.8
5022	29-juin-14	F		59	59	0	36.4	10.4
5024	29-juin-14	F	4		48	0	37.0	11.3
5025	29-juin-14	M	3		36	0	36.0	10.3
5026	29-juin-14	F	3		36	1	37.6	10.1
5027	29-juin-14	M	1		12	0	36.1	10.7
5028	29-juin-14	M	1		12	0	36.1	11.3
5029	29-juin-14	M	1	7	19	0	36.9	10.3
5030	29-juin-14	M	4	10	58	1	36.7	9.4
5031	29-juin-14	M	1		12	0	36.9	9.8
5032	29-juin-14	F	4		48	0	37.0	12.6
5033	29-juin-14	M	4		48	0	36.4	9.7
5034	29-juin-14	F	3		36	0	36.7	11.7
5035	29-juin-14	M	2		24	0	37.1	11.6
5036	29-juin-14	M	2		24	1	36.7	11.5
5038	29-juin-14	F		59	59	1	38.0	10.3
5039	29-juin-14	F	4		48	0	36.1	11.7
5040	29-juin-14	F	3		36	0	35.9	10.7
5041	29-juin-14	F		4	4	0	36.4	10.3
5042	29-juin-14	M	3		36	0	37.1	11.8
5043	29-juin-14	M		8	8	0	36.1	10.7
5044	29-juin-14	M	4		48	0	36.2	10.4
5045	29-juin-14	F	2		24	1	37.0	9.3
5046	29-juin-14	M	3		36	0	37.4	11.4
5047	29-juin-14	M	2		24	0	36.7	10.8
5048	29-juin-14	F		9	9	0	36.8	9.9
5049	29-juin-14	F		59	59	1	36.1	11.6

5050	29-juin-14	M	4		48	0	36.9	11.5
5051	29-juin-14	M	3		36	0	36.7	11.3
5052	29-juin-14	F		6	6	0	37.4	9.6
5053	29-juin-14	F		18	18	0	36.9	11.5
5054	29-juin-14	M	3		36	0	36.8	11.4
5055	29-juin-14	F	2		24	0	36.8	9.4
5056	29-juin-14	M	4		48	0	36.6	11.4
5057	29-juin-14	F	4		48	0	37.7	11.3
5058	29-juin-14	M	2		24	0	36.9	11.9
5059	29-juin-14	M	1	10	22	0	36.3	11.3
5060	29-juin-14	F		41	41	1	36.5	11.4
5061	29-juin-14	F	2		24	0	36.8	11.4
5062	29-juin-14	F	2		24	0	36.5	12.0
5063	29-juin-14	M	4		48	0	36.7	10.2
5064	29-juin-14	F	4		48	0	36.2	10.7
5065	29-juin-14	M		59	59	0	36.1	12.2
5066	29-juin-14	F	3		36	0	36.4	12.3
5067	29-juin-14	M		6	6	0	36.7	11.4
5068	29-juin-14	M	4		48	0	36.8	12.3
5069	29-juin-14	M	4		48	0	36.6	12.6
6001	26-juin-14	F	3		36	0	36.3	10.8
6002	26-juin-14	M	3		36	0	36.8	10.2
6003	26-juin-14	M	3		36	0	36.6	10.2
6004	26-juin-14	F		3	3	0	36.1	12.7
6005	26-juin-14	F	3		36	1	36.1	11.9
6006	26-juin-14	F	3		36	1	36.1	11.2
6007	26-juin-14	M	2		24	0	36.7	8.7
6008	26-juin-14	M	4		48	1	36.7	10.6
6009	26-juin-14	F	2		24	0	36.3	11.2
6010	26-juin-14	F	3		36	0	36.1	11.9
6011	26-juin-14	M		59	59	0	36.4	12.8
6012	26-juin-14	M		59	59	1	36.7	11.9

6013	26-juin-14	M		59	59	0	36.9	11.0
6014	26-juin-14	F		20	20	0	36.5	12.1
6015	26-juin-14	M		59	59	0	37.3	11.8
6016	26-juin-14	F	3		36	0	36.2	10.6
6017	26-juin-14	F		16	16	1	36.8	10.7
6018	26-juin-14	F	4		48	0	36.2	10.5
6019	26-juin-14	F		59	59	0	36.7	10.3
6020	26-juin-14	M		59	59	0	37.2	12.4
6021	26-juin-14	M	3		36	0	36.5	12.2
6022	26-juin-14	M		59	59	0	36.8	13.8
6023	26-juin-14	M	4		48	0	36.9	11.8
6024	26-juin-14	M		59	59	0	37.0	12.6
6025	26-juin-14	M	3		36	0	36.5	11.1
6026	26-juin-14	F		59	59	0	37.3	13.5
6027	26-juin-14	M	3		36	0	36.3	12.9
6028	26-juin-14	M	4		48	0	36.8	10.5
6029	26-juin-14	F	1		12	0	36.1	11.4
6030	26-juin-14	F	3		36	0	36.1	11.7
6031	26-juin-14	M		59	59	0	36.4	11.3
6032	26-juin-14	F	2	6	30	0	36.3	13.6
6033	26-juin-14	M	2		24	0	36.5	9.6
6034	26-juin-14	M	2		24	0	36.5	7.5
6035	26-juin-14	M		8	8	0	36.3	10.5
6036	26-juin-14	M		4	4	0	36.2	11.9
6037	26-juin-14	F		59	59	0	36.9	9.5
6038	26-juin-14	F	2		24	0	36.6	11.4
6039	26-juin-14	F		59	59	0	36.1	11.0
6040	26-juin-14	M	4		48	0	36.7	12.2
6041	26-juin-14	M	3		36	0	36.6	11.1
6042	26-juin-14	M	4		48	0	37.4	10.9
6043	26-juin-14	M		59	59	0	37.0	12.8
6044	26-juin-14	M		4	4	0	36.3	9.6

6045	26-juin-14	M	2		24	0	37.1	10.7
6046	26-juin-14	F	3		36	0	36.2	9.6
6047	26-juin-14	F	4		48	0	36.8	10.5
6048	26-juin-14	M		20	20	0	36.9	11.6
6049	26-juin-14	F	4		48	1	36.7	12.4
6050	26-juin-14	F		59	59	0	36.8	11.2
6051	26-juin-14	F	4		48	1	37.0	11.5
6052	26-juin-14	F	4		48	1	36.9	13.7
6053	26-juin-14	F	2		24	0	37.1	9.5
6055	26-juin-14	M		34	34	0	37.4	9.4
6056	26-juin-14	M		59	59	0	37.1	12.1
6057	26-juin-14	M	3		36	0	37.0	9.9
6058	26-juin-14	M		59	59	0	37.4	12.0
6059	26-juin-14	M		10	10	0	36.9	10.5
6060	26-juin-14	F	3		36	0	36.7	11.0
6061	26-juin-14	F	4		48	0	37.0	11.2
6062	26-juin-14	M	3		36	0	36.9	11.6
6063	26-juin-14	M		11	11	1	36.4	10.8
6064	26-juin-14	F		19	19	0	37.1	10.2
6065	26-juin-14	M		59	59	0	37.0	11.2
6066	26-juin-14	M	3		36	1	39.2	10.5
7001	30-juin-14	F	4		48	1	35.6	10.6
7002	30-juin-14	M	2		24	0	35.5	9.3
7003	30-juin-14	M		4	4	0	36.5	9.5
7004	30-juin-14	F	4		48	0	35.9	11.3
7005	30-juin-14	M	3		36	0	36.2	9.7
7006	30-juin-14	F	3		36	0	36.5	9.6
7007	30-juin-14	M	3		36	1	35.8	9.6
7008	30-juin-14	F	1	6	18	0	36.8	10.7
7009	30-juin-14	F	1		12	0	35.9	10.1
7010	30-juin-14	F		3	3	0	36.0	12.2
7011	30-juin-14	M		59	59	1	35.8	10.9

7012	30-juin-14	F	3		36	0	36.2	10.5
7013	30-juin-14	M		6	6	0	35.6	10.4
7014	30-juin-14	F	3		36	1	36.1	12.0
7015	30-juin-14	F	2		24	0	35.7	11.8
7016	30-juin-14	M	1		12	0	36.0	9.9
7017	30-juin-14	M	3		36	0	36.7	10.3
7018	30-juin-14	M	4		48	0	36.1	12.4
7019	30-juin-14	M	4		48	1	36.0	10.3
7020	30-juin-14	F	3		36	0	37.0	10.5
7021	30-juin-14	M	4		48	0	36.7	10.0
7022	30-juin-14	F	3		36	1	36.1	6.9
7023	30-juin-14	F	2		24	0	36.2	11.3
7024	30-juin-14	F	1		12	0	36.2	6.7
7025	30-juin-14	M	4		48	0	36.4	12.7
7026	30-juin-14	F	3		36	0	36.8	11.7
7027	30-juin-14	F	2		24	0	36.6	10.7
7028	30-juin-14	F	1		12	0	36.2	10.2
7029	30-juin-14	M	3		36	0	36.4	10.9
7030	30-juin-14	M		30	30	0	36.8	11.2
7031	30-juin-14	M	4		48	0	36.8	12.8
7032	30-juin-14	F		59	59	0	36.9	11.5
7033	30-juin-14	F	2		24	0	35.8	11.0
7034	30-juin-14	F	4		48	0	36.6	10.2
7035	30-juin-14	F	2		24	0	36.7	11.7
7037	30-juin-14	M	2		24	0	36.9	10.7
7038	30-juin-14	M	2		24	0	36.1	10.9
7039	30-juin-14	M		10	10	0	36.2	8.3
7040	30-juin-14	F	4		48	0	36.3	11.9
7041	30-juin-14	M			0	0	35.9	10.7
7042	30-juin-14	F		7	7	0	36.4	10.7
7043	30-juin-14	M	4		48	0	36.1	10.4
7044	30-juin-14	M	2		24	0	37.1	10.8

7045	30-juin-14	F	1		12	0	36.8	10.7
7046	30-juin-14	F	2		24	0	36.6	11.4
7047	30-juin-14	F	3		36	0	36.1	10.5
7048	30-juin-14	M	3		36	0	36.7	12.4
7049	30-juin-14	F		10	10	0	36.2	10.0
7050	30-juin-14	F	4		48	0	36.1	10.3
7051	30-juin-14	M		59	59	0	36.1	9.9
7052	30-juin-14	F	1		12	0	36.7	6.0
7053	30-juin-14	F	1		12	0	36.2	8.8
7054	30-juin-14	M		30	30	1	36.9	11.0
7055	30-juin-14	M	3		36	0	36.8	11.3
7056	30-juin-14	M		30	30	0	36.4	11.2
7057	30-juin-14	M		10	10	0	36.1	11.9
7058	30-juin-14	F	4		48	0	36.2	12.4
7059	30-juin-14	F	2		24	0	36.2	11.3
7060	30-juin-14	F	2		24	0	36.1	10.8
7061	30-juin-14	M		59	59	0	36.2	10.9
7062	30-juin-14	M	2		24	1	36.7	8.7
7063	30-juin-14	F		59	59	0	36.7	11.8
7064	30-juin-14	F	3		36	0	36.2	9.9
7065	30-juin-14	M	2		24	0	36.6	10.3
7066	30-juin-14	M	2		24	0	36.4	10.8
7067	30-juin-14	F	2		24	0	36.2	11.8
7068	30-juin-14	M	4		48	0	36.5	11.7
7069	30-juin-14	M	3		36	1	36.1	12.5
7070	30-juin-14	F	3		36	0	36.7	10.9
7071	30-juin-14	F	2		24	0	36.7	10.1
8001	23-juin-14	M	2		24	0	36.8	7.0
8002	23-juin-14	F	4		48	1	37.0	11.0
8003	23-juin-14	M		59	59	0	36.7	10.5
8004	23-juin-14	F	1		12	1	36.2	9.6
8005	23-juin-14	M	3		36	0	37.4	11.2

8006	23-juin-14	M	3		36	0	37.0	10.3
8007	23-juin-14	F	2		24	0	36.6	9.9
8008	23-juin-14	F	4		48	0	36.4	11.3
8009	23-juin-14	M	4		48	0	36.7	11.9
8010	23-juin-14	F	3		36	0	36.9	10.1
8011	23-juin-14	F	2		24	0	36.7	8.4
8012	23-juin-14	F		6	6	0	36.2	10.3
8013	23-juin-14	F	1		12	0	36.4	10.2
8014	23-juin-14	F	1	6	18	0	36.2	10.8
8015	23-juin-14	M	4		48	0	37.0	10.5
8016	23-juin-14	M	4		48	0	36.3	10.6
8017	23-juin-14	F	1		12	0	35.6	9.3
8018	23-juin-14	F	2		24	0	36.2	11.9
8019	23-juin-14	M	4		48	1	38.7	7.6
8020	23-juin-14	F	4		48	0	36.1	12.1
8021	23-juin-14	F	3		36	0	36.4	10.3
8022	23-juin-14	F	3		36	0	36.1	9.3
8023	23-juin-14	F	4		48	0	11.5	11.5
8024	23-juin-14	M	1		12	1	36.7	9.7
8025	23-juin-14	F	2		24	0	36.3	11.0
8026	23-juin-14	M		11	11	0	36.7	10.0
8027	23-juin-14	M	3		36	0	35.7	10.8
8028	23-juin-14	F	3		36	0	36.2	11.6
8029	23-juin-14	F	2		24	0	36.8	10.8
8030	23-juin-14	F		3	3	0	36.7	10.1
8031	23-juin-14	M	3		36	0	36.7	11.8
8032	23-juin-14	M	2		24	0	36.1	9.0
8033	23-juin-14	M	2		24	0	36.7	10.1
8034	23-juin-14	M	3		36	0	36.1	11.2
8035	23-juin-14	M	1		12	0	36.7	10.9
8036	23-juin-14	M	2		24	0	37.0	12.3
8037	23-juin-14	M	4		48	1	36.8	10.1

8038	23-juin-14	F	3		36	0	36.3	11.8
8039	23-juin-14	F	3		36	0	36.3	10.5
8040	23-juin-14	M	1		12	1	37.4	10.4
8041	23-juin-14	F	3		36	0	36.4	12.3
8042	23-juin-14	M	2	6	30	1	36.7	11.0
8043	23-juin-14	M	4		48	0	37.4	13.0
8045	23-juin-14	M		5	5	0	37.1	9.2
8046	23-juin-14	M		5	5	0	36.9	8.3
8047	23-juin-14	F		59	59	0	36.7	12.2
8048	23-juin-14	F		59	59	0	37.4	12.3
8049	23-juin-14	M	4		48	1	37.0	11.2
8050	23-juin-14	F	3		36	0	36.6	11.4
8051	23-juin-14	F	3		36	0	36.9	10.0
8052	23-juin-14	F	3		36	0	36.9	10.6
8053	23-juin-14	F	2		24	0	37.4	9.3
8054	23-juin-14	F	2		24	0	36.4	11.0
8055	23-juin-14	M	2	3	27	0	36.2	11.2
8056	23-juin-14	M		3	3	0	36.9	10.4
8057	23-juin-14	M	1		12	0	37.1	11.0
8058	23-juin-14	M	3		36	0	37.0	11.3
8059	23-juin-14	F		59	59	0	37.1	10.0
8060	23-juin-14	M	3		36	1	37.0	9.7
8061	23-juin-14	M		12	12	0	36.1	10.5
8062	23-juin-14	M	1	3	15	0	37.0	10.3
8063	23-juin-14	F		59	59	0	36.7	13.3
8064	23-juin-14	F	4		48	0	36.5	11.5
8065	23-juin-14	F	2		24	1	36.8	10.0
8066	23-juin-14	M	1		12	0	37.2	9.5
8067	23-juin-14	M	3		36	1	37.1	9.9
8068	23-juin-14	M		17	17	0	36.7	10.9
8069	23-juin-14	F		59	59	0	37.3	12.0
9001	27-juin-14	M	4		48	0	36.0	10.0

9002	27-juin-14	F	2		24	1	36.1	7.6
9003	27-juin-14	F	3		36	0	35.7	11.8
9004	27-juin-14	F	2		24	1	36.3	10.6
9005	27-juin-14	F	4		48	0	36.5	11.1
9006	27-juin-14	F	2		24	0	37.4	11.8
9008	27-juin-14	F	2	7	31	0	37.5	9.9
9009	27-juin-14	M		59	59	0	36.1	8.8
9010	27-juin-14	F	4	6	54	0	36.5	11.3
9011	27-juin-14	F		4	4	0	36.2	11.1
9013	27-juin-14	M	3		36	0	37.0	10.8
9015	27-juin-14	F		59	59	0	35.9	12.4
9016	27-juin-14	M		59	59	0	36.9	12.8
9017	27-juin-14	F		59	59	0	35.9	12.2
9018	27-juin-14	M		7	7	0	36.5	11.0
9019	27-juin-14	F		56	56	0	35.9	13.9
9020	27-juin-14	F		59	59	0	36.1	11.2
9021	27-juin-14	M	1	1	13	0	36.2	8.4
9022	27-juin-14	F		59	59	0	36.8	11.5
9023	27-juin-14	M	3		36	0	36.4	9.9
9024	27-juin-14	M		59	59	1	37.3	9.9
9025	27-juin-14	M		59	59	0	37.3	11.3
9026	27-juin-14	F	4		48	0	36.8	12.0
9027	27-juin-14	M	1		12	0	36.8	9.3
9028	27-juin-14	M	2	6	30	1	39.1	10.9
9029	27-juin-14	F	3		36	1	36.1	11.3
9030	27-juin-14	M	4		48	1	36.7	10.1
9031	27-juin-14	F		59	59	1	36.1	12.1
9032	27-juin-14	M	3		36	0	36.9	11.8
9033	27-juin-14	F	4		48	0	37.1	11.9
9034	27-juin-14	M	1		12	0	37.2	8.2
9035	27-juin-14	M	1		12	0	36.8	11.6
9036	27-juin-14	M		59	59	0	36.2	11.1

9037	27-juin-14	M	4		48	0	36.7	10.0
9038	27-juin-14	F	4		48	0	36.9	11.1
9039	27-juin-14	M		18	18	0	36.7	12.9
9040	27-juin-14	M	4		48	0	37.0	12.0
9041	27-juin-14	F	4		48	0	36.9	12.3
9042	27-juin-14	F		59	59	0	37.6	11.2
9043	27-juin-14	M	3		36	1	39.1	13.0
9044	27-juin-14	M	3		36	0	36.2	10.1
9045	27-juin-14	M	4		48	0	36.9	11.0
9046	27-juin-14	F	2		24	0	36.8	9.0
9048	27-juin-14	M		48	48	1	37.0	11.6
9049	27-juin-14	F	1	9	21	0	36.9	12.5
9050	27-juin-14	F	3		36	0	36.4	10.0
9051	27-juin-14	M		59	59	0	36.7	11.2
9052	27-juin-14	M	4		48	0	36.7	11.9
9053	27-juin-14	F		59	59	0	37.5	10.0
9054	27-juin-14	M	3		36	0	37.5	10.4
9055	27-juin-14	M		59	59	0	36.8	11.1
9056	27-juin-14	F	2		24	0	36.9	10.9
9057	27-juin-14	M	2		24	0	36.7	10.6
9058	27-juin-14	M	3	6	42	0	36.9	11.0
9059	27-juin-14	M	3		36	0	37.1	12.0
9060	27-juin-14	F		59	59	1	36.9	11.7
9061	27-juin-14	F	3		36	0	37.1	12.8
9062	27-juin-14	F		14	14	1	36.7	8.2
9063	27-juin-14	F		9	9	0	36.9	10.0
9064	27-juin-14	M	4		48	1	36.8	11.2
9065	27-juin-14	F	2		24	1	37.0	13.0
9066	27-juin-14	M	1	6	18	0	36.6	10.3
9067	27-juin-14	F	2		24	0	36.9	11.2
9068	27-juin-14	F		59	59	0	36.4	11.6

RDT Result
0
0
1
1
0
0
0

0
1
1

1

0
0
0

0
0
1
0
0
0
0

0
0
0
0
1
1
0

1
1
1
0

0
0
0

1

0
0
0
1
0
0

1
0
0

0
0
0
0
1
1
1

0
1
0

0
0
1
0

0
0
1

0
0
1
0
0
0

1
0
0
1
0
0
0

1
1
0
0
1
1
