# Supplementary material for: Seasonal Malaria Chemoprevention with Sulphadoxine-Pyrimethamine and Amodiaquine Selects Pfdhfr-dhps Quintuple Mutant Genotype in Mali
Source: PLoS One. 2016 Sep 23;11(9):e0162718. doi: 10.1371/journal.pone.0162718 (PMC5035027; doi:10.1371/journal.pone.0162718)
Supplement: S4 File — (PDF) [file pone.0162718.s004.pdf]

ID	*P.falciparum (Trophozoites/microliter)*	*P.malariae (Trophozoites/microliter)*	*Pfdhfr164*	*Pfdhps540*	*Pfdhps437*	*Pfdhfr51*	*Pfdhfr59*	*Pfdhfr108*	*Pfcrt-75*
1165	3320	0	1	1	2	2	2	2	2
8001	440	0	1	1	2	2	2	2	2
8002	640	0	1	1	2	2	2	2	2
8003	6640	0	1	1	2	2	2	2	2
8005	7000	0	1	1	1	2	2	2	2
8007	5880	0	1	1	1	2	2	2	1
8010	2640	0	1	1	2	2	2	2	2
8015	3080	0	1	1	2	2	2	2	2
8016	5840	0	1	1	1	2	2	2	2
8017	360	0	1	1	2	2	2	2	2
8027	1800	0	1	1	2	2	2	2	2
8032	0	2880	1	2	2	2	2	2	2
8037	600	0	1	1	2	2	2	2	2
8038	1080	0	1	1	2	2	2	2	2
8047	440	0	1	1	2	2	2	2	2
8048	240	0	1	1	2	2	2	2	2
8049	200	0	1	1	2	2	2	2	2
8055	23680	0	1	1	2	2	2	2	1
8059	2240	0	1	2	1	2	2	2	2
8063	80	0	1	1	1	2	2	2	1
1158	200	0	1	1	1	2	2	2	1
1155	480	0	1	1	1	2	2	2	1
1154	160	0	1	1	2	2	2	2	2
1148	280	0	1	1	2	2	2	2	2
1147	960	0	1	1	2	2	2	2	1
1141	320	0	1	2	2	2	2	2	2

1139	560	0	1	1	2	2	2	2	2
1138	12720	0	1	1	2	2	2	2	1
1137	560	0	1	1	2	2	2	2	1
1120	1280	0	1	1	2	2	2	2	2
1119	1040	0	1	1	2	2	2	2	1
1118	12720	0	1	1	2	2	2	2	1
1115	320	0	1	1	2	2	2	2	1
1114	13600	0	1	1	2	2	2	2	2
1112	680	0	1	1	2	2	2	2	1
1110	240	0	1	2	2	2	2	2	2
6063	80	0	1	1	2	2	2	2	2
6061	1280	0	1	1	1	2	2	2	2
2067	1120	0	1	1	1	2	2	2	2
2056	1160	0	1	1	2	2	2	2	2
2036	560	120	1	1	2	2	2	2	2
2034	2080	0	1	1	2	2	2	2	2
2030	80	0	1	2	2	2	2	2	2
2023	1760	0	1	1	2	2	2	2	2
2021	1040	0	1	1	2	2	2	2	2
2018	400	0	1	1	2	2	2	2	2
2015	0	880	1	2	1	2	2	2	2
2011	120	0	1	1	1	2	2	2	2
2010	200	0	1	1	2	2	2	2	2
2007	80	0	1	2	2	2	2	2	2
2006	200	0	1	1	1	2	2	2	2
2005	400	0	1	1	1	2	2	2	2
2004	1240	0	1	1	2	2	2	2	2
2001	1480	0	1	1	1	2	2	2	1
5006	7800	0	1	1	2	2	2	2	1
5020	3400	0	1	1	2	2	2	2	2
5025	3840	0	1	1	2	2	2	2	1
5038	120	0	1	1	2	2	2	2	2

5063	760	0	1	1	2	2	2	2	1
7063	120	0	1	1	2	2	2	2	2
7032	200	0	1	1	2	2	2	2	2
7028	840	0	1	1	1	2	2	2	2
7020	160	0	1	1	2	2	2	2	2
7019	200	0	1	1	1	2	2	2	2
7014	160	0	1	1	1	2	2	2	2
3055	1280	0	1	1	2	2	2	2	2
3054	360	0	1	1	2	2	2	2	2
3046	920	0	1	1	2	2	2	2	2
3045	440	0	1	1	1	2	2	2	2
3043	360	0	1	1	2	2	2	2	2
1080	1240	0	1	1	1	2	2	2	2
1070	200	0	1	1	1	2	2	2	2
1069	6880	0	1	1	1	2	2	2	2
1067	256	0	1	1	1	2	2	2	2
1065	680	0	1	1	2	2	2	2	1
1064	960	0	1	1	2	2	2	2	2
1050	4400	0	1	1	2	2	2	2	1
1040	400	0	1	1	2	2	2	2	2
1031	120	0	1	1	2	2	2	2	2
1028	3640	0	1	2	2	2	2	2	2
1015	114520	0	1	1	1	2	2	2	1
1014	480	0	1	1	1	2	2	2	1
1007	640	0	1	2	1	2	2	2	1
1006	3720	0	1	1	2	2	2	2	2
1001	360	0	1	1	2	2	2	2	2

Pfmdr1-86
1
1
1
2
1
1
1
1
1
1
1
1
1
1
2
1
1
2
1
1
1
1
1
1
2
2

1
1
1
1
2
1
1
2
2
1
1
1
2
1
2
1
1
1
1
1
1
2
1
1
1
1
1
1
1
1
1
1

1
1
1
1
1
1
1
1
1
1
1
1
1
2
2
1
1
1
1
1
1
1
1
1
1
1
1
