# Supplementary material for: Seasonal Malaria Chemoprevention with Sulphadoxine-Pyrimethamine and Amodiaquine Selects Pfdhfr-dhps Quintuple Mutant Genotype in Mali
Source: PLoS One. 2016 Sep 23;11(9):e0162718. doi: 10.1371/journal.pone.0162718 (PMC5035027; doi:10.1371/journal.pone.0162718)
Supplement: S5 File — (PDF) [file pone.0162718.s005.pdf]

ID	SEX	Age/year	RDT_Result
1001	F	80	1
1002	F	13	1
1003	F	10	1
1004	M	73	2
1005	M	11	1
1006	F	8	1
1007	M	24	1
1008	F	12	2
1009	F	17	1
1010	M	28	1
1011	F	18	2
1012	F	24	2
1013	F	39	1
1014	M	17	1
1015	F	30	1
1016	F	20	1
1017	F	30	1
1018	M	20	2
1019	F	18	1
1020	F	20	2
1021	F	40	2
1022	F	28	2
1023	F	35	2
1024	F	25	1
1025	M	24	2
1026	F	37	2
1027	F	23	1
1028	F	26	2
1029	M	49	1

1030	F	10	1
1031	F	10	1
1032	M	14	2
1033	M	11	1
1034	F	46	2
1035	F	8	1
1036	F	21	1
1037	F	45	1
1038	F	28	1
1039	F	8	2
1040	F	29	2
1041	M	15	1
1042	M	8	2
1043	M	36	1
1044	F	32	2
1045	M	33	1
1046	F	30	2
1047	F	75	2
1048	M	36	1
1049	F	50	2
1050	F	18	1
2001	F	20	1
2002	M	14	1
2003	F	9	1
2004	M	15	1
2005	M	8	1
2006	M	16	1
2007	M	46	1
2008	M	14	1
2009	M	30	1

2010	M	17	1
2011	F	20	1
2012	M	12	1
2013	F	12	1
2014	M	14	1
2015	M	16	1
2016	F	18	1
2017	F	8	1
2018	M	15	1
2019	F	13	1
2020	M	12	1
2021	F	8	1
2022	M	8	1
2023	M	11	2
2024	F	17	2
2025	M	48	1
2026	M	12	1
2027	F	45	1
2028	F	32	1
2029	M	10	1
2030	F	8	1
2031	F	30	1
2032	M	10	1
2033	F	12	1
2034	M	15	1
2035	M	12	1
2036	F	48	1
2037	F	20	2
2038	F	11	1
2039	M	11	1

2040	F	21	2
2041	F	19	2
2042	F	18	1
2043	M	15	1
2044	F	20	1
2045	M	17	1
2046	F	19	1
2047	F	9	1
2048	F	24	1
2049	F	12	1
2050	M	9	1
3001	M	14	1
3002	F	11	1
3003	M	26	2
3004	F	13	2
3005	F	14	1
3006	M	8	1
3007	M	32	1
3008	F	16	1
3009	M	18	1
3010	F	8	1
3011	F	30	1
3012	F	14	1
3013	M	8	1
3014	F	14	1
3015	F	35	2
3016	F	24	1
3017	M	13	1
3018	M	15	1
3019	M	17	2

3020	M	23	1
3021	F	30	1
3022	F	35	1
3023	F	80	1
3024	M	16	1
3025	M	8	1
3026	F	20	1
3027	M	16	1
3028	F	34	2
3029	F	12	1
3030	M	14	1
3031	F	19	1
3032	F	13	1
3033	F	13	2
3034	M	8	1
3035	M	39	1
3036	M	13	1
3037	M	12	1
3038	M	8	2
3039	F	30	1
3040	M	14	1
3041	M	8	1
3042	M	40	1
3043	F	60	1
3044	M	14	1
3045	F	12	1
3046	F	26	2
3047	M	12	1
3048	M	10	1
3049	F	54	1

3050	F	8	1
4001	F	8	1
4002	F	23	1
4003	F	25	1
4004	M	8	1
4005	M	9	2
4006	F	9	1
4007	F	20	1
4008	M	20	1
4009	F	18	2
4010	M	19	2
4011	M	24	2
4012	F	25	1
4013	F	10	2
4014	F	25	1
4015	F	20	2
4016	F	19	2
4017	F	24	1
4018	F	24	1
4019	M	19	1
4020	F	9	1
4021	F	15	1
4022	M	8	1
4023	F	24	1
4024	F	23	1
4025	F	30	2
4026	F	18	2
4027	M	18	2
4028	F	14	2
4029	F	15	1

4030	M	8	1
4031	F	25	2
4032	M	8	2
4033	M	24	2
4034	F	23	1
4035	F	13	1
4036	F	20	1
4037	F	12	2
4038	F	18	2
4039	M	14	2
4040	F	24	2
4041	F	29	1
4042	F	21	1
4043	F	14	2
4044	F	22	2
4045	F	25	1
4046	F	12	1
4047	F	21	2
4048	M	8	1
4049	F	25	1
4050	F	25	2
5001	F	35	2
5002	F	30	1
5003	F	40	1
5004	M	30	1
5005	M	9	1
5006	F	25	1
5007	F	30	1
5008	F	30	1
5009	M	14	1

5010	M	32	1
5011	F	22	1
5012	F	20	2
5013	F	16	1
5014	M	8	1
5015	F	19	1
5016	F	27	1
5017	F	20	1
5018	F	25	1
5019	F	35	1
5020	F	13	1
5021	F	36	1
5022	F	50	1
5023	F	26	2
5024	M	37	2
5025	M	8	1
5026	M	21	1
5027	M	22	1
5028	F	30	1
5029	F	8	1
5030	M	11	2
5031	F	25	1
5032	F	18	1
5033	F	13	1
5034	F	20	1
5035	F	37	1
5036	F	22	1
5037	F	19	2
5038	F	56	2
5039	F	19	2

5040	F	60	2
5041	F	35	1
5042	F	20	1
5043	F	35	1
5044	F	40	1
5045	F	60	2
5046	F	12	1
5047	F	29	1
5048	M	11	1
5049	F	25	1
5050	F	28	1
5051	F	9	1
6001	M	13	2
6002	M	48	1
6003	F	28	2
6004	M	8	1
6005	F	12	1
6006	F	40	2
6007	M	8	1
6008	F	37	2
6009	F	33	2
6010	M	35	2
6011	M	35	2
6012	F	8	1
6013	M	13	1
6014	M	33	1
6015	F	35	1
6016	F	39	1
6017	M	10	1
6018	F	36	2

6019	M	59	1
6020	F	8	1
6021	F	38	2
6022	F	8	1
6023	F	9	1
6024	F	29	1
6025	F	12	1
6026	F	14	1
6027	M	45	1
6028	F	12	1
6029	M	32	1
6030	F	8	1
6031	F	28	1
6032	F	52	1
6033	M	16	2
6034	F	13	1
6035	F	25	2
6036	M	14	1
6037	F	41	1
6038	M	43	1
6039	F	8	1
6040	F	60	1
6041	M	40	1
6042	M	11	1
6043	F	30	2
6044	M	8	1
6045	M	42	2
6046	M	37	2
6047	F	55	2
6048	M	13	1

6049	F	30	1
6050	F	8	1
7001	M	45	2
7002	M	50	2
7003	F	23	2
7004	F	44	1
7005	F	14	1
7006	M	12	1
7007	M	16	1
7008	F	27	1
7009	M	24	1
7010	M	20	2
7011	M	22	1
7012	F	17	1
7013	F	53	1
7014	M	45	1
7015	F	8	1
7016	F	25	1
7017	M	18	1
7018	M	28	1
7019	M	31	1
7020	F	20	1
7021	F	15	1
7022	M	49	1
7023	F	44	2
7024	M	36	2
7025	F	60	1
7026	F	12	1
7027	F	50	2
7028	F	34	2

7029	F	43	2
7030	F	42	1
7031	F	31	1
7032	F	19	1
7033	M	19	1
7034	F	14	1
7035	F	25	1
7036	F	24	1
7037	F	32	1
7038	M	26	2
7039	F	64	2
7040	F	27	1
7041	M	29	1
7042	F	25	1
7043	F	20	1
7044	M	24	2
7045	M	36	1
7046	F	27	1
7047	F	22	2
7048	M	26	2
7049	F	20	2
7050	F	8	1
7051	M	54	1
8001	M	20	2
8002	F	36	1
8003	F	13	1
8004	F	28	2
8005	M	23	2
8006	F	33	1
8007	F	54	1

8008	M	32	2
8009	F	23	1
8010	F	24	1
8011	F	23	1
8012	F	23	2
8013	F	50	1
8014	F	38	2
8015	F	33	1
8016	F	20	1
8017	F	25	1
8018	F	58	1
8019	F	35	1
8020	F	60	2
8021	F	35	1
8022	F	34	1
8023	M	40	1
8024	F	30	2
8025	F	38	2
8026	F	17	1
8027	M	37	1
8028	F	32	2
8029	F	49	1
8030	M	31	1
8031	F	35	1
8032	M	34	2
8033	F	45	1
8034	M	25	1
8035	F	36	1
8036	F	28	1
8037	F	33	1

8038	F	19	2
8039	F	26	1
8040	F	31	2
8041	F	19	1
8042	M	18	2
8043	F	57	2
8044	F	36	1
8045	M	16	2
8046	F	14	1
8047	M	55	1
8048	F	16	1
8049	F	24	1
8050	F	18	1
9001	M	8	1
9002	F	8	1
9003	M	8	1
9004	F	8	1
9005	M	45	2
9006	F	8	2
9007	M	8	1
9008	M	22	1
9009	F	23	1
9010	F	9	1
9011	M	9	1
9012	F	14	2
9013	F	12	1
9014	M	9	1
9015	M	10	1
9016	F	10	1
9017	M	12	1

9018	F	12	2
9019	M	26	1
9020	M	47	1
9021	M	8	1
9022	F	8	1
9023	F	12	1
9024	F	13	1
9025	M	9	1
9026	M	8	1
9027	F	9	1
9028	F	9	1
9029	M	8	1
9030	M	41	1
9031	M	14	1
9032	F	10	1
9033	F	12	2
9034	F	13	1
9035	F	8	1
9036	M	11	2
9037	F	14	1
9038	M	44	1
9039	M	10	1
9040	M	8	2
9041	M	8	1
9042	F	9	2
9043	M	8	2
9044	F	8	2
9045	M	9	1
9046	F	9	1
9047	M	8	1

9048	M	9	2
9049	M	9	1
9050	F	8	2
1101	M	40	1
1102	M	37	1
1103	F	15	1
1104	M	39	2
1105	M	91	1
1106	F	36	1
1107	M	57	2
1108	F	35	2
1109	M	25	1
1110	M	9	1
1111	M	12	1
1112	F	19	1
1113	F	25	2
1114	F	31	2
1115	M	54	1
1116	M	28	1
1117	M	37	2
1118	M	32	1
1119	M	8	1
1120	M	20	2
1121	F	20	2
1122	M	24	1
1123	M	12	1
1124	M	41	2
1125	M	21	1
1126	M	43	1
1127	M	34	2

1128	M	8	1
1129	F	45	2
1130	M	32	2
1131	F	20	2
1132	M	12	1
1133	M	42	1
1134	F	8	1
1135	M	14	1
1136	F	8	1
1137	F	32	1
1138	F	22	2
1139	M	24	2
1140	M	53	1
1141	F	30	2
1142	M	8	1
1143	F	22	1
1144	F	19	1
1145	F	20	1
1146	F	28	2
1147	F	23	1
1148	M	37	2
1149	M	46	2
1150	F	25	1
