# Supplementary material for: Seasonal Malaria Chemoprevention with Sulphadoxine-Pyrimethamine and Amodiaquine Selects Pfdhfr-dhps Quintuple Mutant Genotype in Mali
Source: PLoS One. 2016 Sep 23;11(9):e0162718. doi: 10.1371/journal.pone.0162718 (PMC5035027; doi:10.1371/journal.pone.0162718)
Supplement: S6 File — (PDF) [file pone.0162718.s006.pdf]

ID	*PfDHPS540*	*PfDHPS437*	*PfDHFR108*	*PfDHFR51*	*PfDHFR59*	*PfCRT-75*	*PfMDR1-86*
3032	1	2	2	2	0	0	1
3006	1	2	2	2	2	1	1
3025	1	2	2	2	2	2	2
3030	1	2	2	1	2	2	0
3002	1	1	2	2	2	2	0
3009	1	2	2	2	2	2	0
3017	1	2	2	2	0	2	2
3034	1	2	1	2	1	2	1
3035	1	1	1	1	1	2	1
3016	1	2	2	2	2	1	1
3013	1	2	0	2	2	2	0
3047	1	0	0	0	2	0	0
3027	1	2	2	2	2	0	0
3005	1	2	2	2	2	0	0
3010	1	1	1	2	2	0	0
3050	1	2	2	2	2	0	0
3026	1	2	1	2	2	2	1
3012	1	0	0	0	0	0	0
5022	1	0	0	2	0	0	0
5028	1	2	2	2	2	0	0
5007	1	2	1	1	1	2	0
5010	1	2	2	2	2	2	2
5031	1	2	2	2	2	0	0
5008	1	2	2	2	2	2	1
5014	1	1	2	2	2	1	1
5042	1	1	2	2	2	1	1
5048	1	2	2	2	2	0	0
5025	1	2	2	2	2	2	2
5043	1	0	0	2	0	0	0

5036	1	1	2	2	0	0	0
5044	1	2	2	1	2	2	0
5020	1	0	0	2	0	0	0
5011	1	0	2	2	2	0	0
5035	1	2	2	2	2	0	1
5009	1	0	2	2	2	0	0
5018	1	2	1	2	2	0	0
8007	1	2	1	1	1	1	0
8044	1	0	2	2	2	0	0
8022	1	0	2	0	0	0	0
8033	1	2	0	2	2	0	0
8009	1	0	0	0	0	0	0
8010	1	2	2	2	2	0	1
8048	1	0	0	0	0	0	0
8027	1	0	0	2	2	0	0
8037	1	1	2	2	2	0	0
8017	1	2	2	1	2	0	1
8036	2	0	2	0	0	0	0
8013	3	2	2	2	2	0	0
8041	3	0	0	0	0	0	0
8047	1	2	1	1	0	0	0
8030	1	2	2	2	2	2	2
8018	1	2	2	2	2	2	2
8011	1	2	2	2	2	2	1
8015	1	1	1	1	0	2	2
8006	1	1	2	2	2	2	0
6037	1	1	0	2	2	0	0
6024	1	1	2	2	2	0	0
6014	1	0	0	2	2	0	0
6040	1	1	1	2	2	0	0

6036	1	1	2	2	2	2	0
6039	1	2	2	2	2	2	1
6028	1	2	2	2	2	2	1
6023	1	2	1	2	1	1	1
6027	1	1	1	2	2	0	0
6048	1	2	2	2	2	0	0
6041	1	2	1	2	1	0	0
6019	1	1	2	2	0	2	0
6030	1	2	2	2	0	2	1
6025	1	2	2	2	0	2	1
6038	1	0	0	2	0	0	0
6013	3	0	0	2	2	0	0
6049	2	1	0	1	2	0	0
6031	2	0	2	2	2	0	0
6002	3	1	2	2	2	0	0
6005	1	1	2	2	2	1	0
6042	1	2	2	2	2	2	0
6020	1	2	2	2	2	2	1
6007	1	2	2	0	2	2	2
6034	1	2	1	0	2	1	1
4036	2	2	2	2	1	2	1
4048	1	2	2	2	2	2	1
4030	1	2	2	2	2	2	0
4003	1	2	1	2	2	2	0
4041	1	2	2	2	2	2	0
4018	1	2	1	1	0	1	0
4036	1	2	0	2	0	2	0
4026	1	1	0	2	1	2	0
4021	1	0	2	2	2	2	0
4045	1	2	2	2	2	2	0

4019	1	0	0	2	2	2	0
4017	1	1	2	2	2	2	0
4042	1	1	2	1	1	2	0
4046	1	0	0	1	0	0	0
4029	1	2	2	1	2	2	0
4014	1	2	2	1	2	1	1
4049	1	2	2	1	2	2	0
4007	1	2	0	2	2	2	0
4009	1	1	0	2	2	2	0
4023	3	1	2	2	2	2	0
4012	1	1	2	2	2	2	1
4022	1	2	2	2	2	1	2
1142	1	0	0	2	2	2	0
1144	1	1	2	2	2	2	2
1112	1	2	2	2	2	1	2
1109	1	2	1	1	2	1	1
1134	1	2	2	2	2	2	2
1123	1	1	2	2	2	1	1
1122	1	0	1	2	2	1	1
1103	1	2	2	2	2	2	1
1147	1	1	2	1	2	1	1
1128	1	1	2	2	0	2	0
2017	1	2	2	2	1	0	0
2025	1	2	2	2	2	0	0
2021	1	2	2	2	2	0	1
2005	1	1	2	2	2	0	1
2012	1	1	1	1	1	0	1
2004	1	2	2	2	2	0	0
2046	1	2	2	2	2	0	0
2016	1	2	2	1	2	0	0

2003	1	2	2	2	2	0	1
2045	1	1	2	1	1	0	0
2022	1	2	2	2	0	1	1
2008	1	2	2	2	2	1	0
2015	1	2	2	2	2	2	2
2030	1	1	2	2	2	1	1
2018	1	2	2	2	2	2	2
2007	1	1	2	1	2	0	1
2035	1	2	2	2	2	2	1
2009	1	2	2	2	2	1	1
2032	1	2	2	2	2	1	1
2047	1	2	2	1	2	2	0
2020	1	1	1	1	1	0	0
2014	1	1	2	2	2	0	0
2015	1	1	1	2	2	0	0
2028	1	2	2	2	2	2	1
2048	1	1	1	1	1	1	1
2034	1	2	2	2	2	2	0
2036	1	1	2	2	2	1	1
2027	1	1	2	2	2	2	1
2002	1	0	0	0	0	0	0
2029	1	1	2	2	2	2	2
2006	1	2	2	1	1	2	2
2031	1	2	1	1	2	0	1
2010	1	1	1	1	1	2	0
2001	1	2	2	2	2	2	1
2026	1	0	2	2	1	2	1
2049	1	2	2	2	2	2	0
9022	1	1	1	1	1	2	0
9046	1	2	2	2	2	2	0

9028	1	2	2	1	2	2	0
9023	1	2	0	1	1	2	0
9034	1	1	2	2	2	1	0
9002	1	2	2	2	2	1	0
9004	1	2	2	2	2	2	0
9013	1	1	2	2	2	2	0
9032	1	1	2	1	2	2	0
9049	1	2	2	2	2	1	0
9011	1	2	1	1	1	0	0
9007	1	1	2	2	0	2	1
9016	1	2	2	2	2	2	0
9001	1	1	2	2	2	0	0
9008	1	0	2	2	2	1	1
9047	1	2	0	1	2	1	1
9037	1	2	2	2	2	1	0
9009	1	0	2	2	0	0	0
9014	1	0	0	2	0	0	0
9038	1	2	1	1	1	1	1
9017	1	0	2	2	2	1	0
9045	1	0	0	2	2	2	0
9031	1	0	0	2	2	2	0
9010	1	0	0	2	2	2	0
9024	1	2	0	2	2	1	2
9026	1	0	0	2	2	0	0
9039	1	0	0	1	2	0	0
9019	1	2	2	2	2	2	0
9015	1	0	0	2	2	0	0
9035	1	0	0	1	1	1	0
9003	1	0	1	1	2	2	1
9041	1	1	2	2	2	1	1

7020	1	2	2	2	2	0	1
7036	1	2	0	2	2	0	0
7019	1	2	2	2	2	1	1
7009	1	2	1	0	2	2	1
7025	1	1	1	0	2	2	2
7016	1	1	2	2	2	2	0
7037	1	1	2	0	2	2	1
7051	1	2	1	0	1	2	0
7018	1	2	2	2	2	2	1
7021	1	2	2	2	2	0	0
7017	1	2	2	2	2	0	1
7040	1	2	2	2	2	0	0
7042	1	2	0	2	2	0	0
7026	1	2	0	2	2	1	1
7008	1	2	2	2	2	0	1
7050	1	2	2	0	2	0	1
7006	1	0	2	2	2	2	1
7032	1	2	1	0	1	0	1
7005	1	0	2	2	2	0	0
1014	1	2	2	0	0	1	1
1045	1	2	2	0	1	0	1
1036	1	1	2	2	2	2	1
1016	1	2	2	2	2	0	1
1030	1	2	2	2	2	2	1
1006	1	2	2	2	2	2	1
1003	1	2	2	2	2	0	2
1050	1	2	0	2	2	0	2
1005	1	2	2	2	2	2	1
1001	1	2	2	2	2	2	1
1041	1	0	0	2	2	2	0

1017	1	2	2	2	2	2	1
1035	1	2	0	2	2	0	0
1043	1	2	2	2	2	2	1
1009	1	2	0	2	2	0	2
1038	1	2	1	2	2	0	0
1013	1	2	2	2	2	1	1
1018	1	2	0	2	2	0	1
1010	1	0	2	2	2	0	1
1029	1	0	2	2	2	0	1
1119	1	0	2	2	2	0	1
1110	1	2	2	2	2	2	0
